# Effective Microorganisms as *Halal*-Based Sources for Biofertilizer Production and Some Socio-Economic Insights: A Review

**DOI:** 10.3390/foods12081702

**Published:** 2023-04-19

**Authors:** Chee Kong Yap, Khalid Awadh Al-Mutairi

**Affiliations:** 1 Department of Biology, Faculty of Science, Universiti Putra Malaysia (UPM), Serdang 43400, Selangor, Malaysia; 2 Department of Biology, Faculty of Science, University of Tabuk, Tabuk P.O. Box 741, Saudi Arabia

**Keywords:** effective microorganisms (EM), *Halal*-based source, biofertilizer, socio-economy

## Abstract

This paper aims to review the literature on ‘Effective Microorganism (EM)’ and ‘Fertilizer’ from the Scopus database and to discuss EMs using *Halal*-based sources for biofertilizer production from socio economic insights. Based on EM and fertilizer publications on the Scopus database, all the 17 papers reviewed provided no detailed information on the *Halal*-status of the biofertilizers inoculated with EM. The impacts of *Halal*-certified biofertilizers will trigger the *Halal* certification in food products by (a) catering for the increasing *Halal* food demand due to expectedly Muslim population expansion, (b) contributing to the sustainable buying behaviour of *Halal* products’ consumers in the future, (c) catering for the increasing number of Muslim travellers around the world, (d) becoming a positive driver for higher production of more *Halal* foods that can enhance food safety, human health and well-being, and (e) creating a cost-effective and increasing food marketability. The later three points (c, d and e) play a very important role in a country’s societal well-being and economic growth and development. Although *Halal*-status is not a must for the world’s food marketing, *Halal*-certified biofertilizer for the *Halal*-status of food carries the greatest potential to enter the ever-expanding Muslim markets. Finally, it is postulated that the successful usage of EM using *Halal*-based sources for biofertilizer production will result in two major outcomes from the points of United Nations’ Sustainable Development Goals # 9 (Industry, Innovation and Infrastructure) and # 12 (Responsible Consumption and Production). Hence, the presented review provides a starting point for future research considering sustainability and innovation as priorities.

## 1. Introduction

### 1.1. The History of Halal Certification in Food Products

*Halal* food is well connected to *Halal* certification. Those food items with *Halal* certification are named as *Halal* foods. The connection between *Halal* certification and food can be easily found in the literature [1,2,3,4,5,6,7,8,9,10,11,12,13,14,15,16,17,18,19,20,21,22,23,24,25,26,27,28,29,30,31,32,33,34,35,36,37,38,39,40,41,42,43,44,45,46,47,48,49,50,51,52,53,54,55,56,57,58,59,60,61,62,63,64,65,66,67,68,69,70,71,72,73,74,75,76,77,78,79,80,81,82,83,84,85,86,87,88,89,90,91,92,93,94,95,96,97,98,99,100,101,102,103,104,105,106,107,108,109,110,111,112,113,114,115,116,117,118,119,120,121,122,123,124,125,126,127,128,129,130,131,132,133,134,135,136,137,138,139,140,141,142,143,144]. Researchers have discussed *Halal* certification from the perspectives of the economy [1,2,3,4,5,6,7,8,9,10,11,12,13,14,15,16,17,18,19,20,21,22,23,24,25,26,27,28,29,30,31,32,33,34,35,36,37,38,39,40,41,42,43,44,45,46,47,48,49,50,51,52,53,54,55,56] in Table S1, the environment [57,58,59,60,61,62,63,64,65,66,67,68,69,70,71,72,73,74,75,76,77,78,79,80,81,82,83,84,85,86,87,88,89] in Table S2, and society [90,91,92,93,94,95,96,97,98,99,100,101,102,103,104,105,106,107,108,109,110,111,112,113,114,115,116,117,118,119,120,121,122,123,124,125,126,127,128,129,130,131,132,133,134,135,136,137,138,139,140,141,142,143,144,145,146,147,148] in Table S3. *Halal* certification ensures that food is permitted or “permissible” for Muslims. Certification also provides opportunities for merchants to establish operational and product differentiation strategies concentrating on other attributes beyond pricing such as convenience, providing variety, consistency, quality, and safety [23]. The concept, definition, or understanding of *Halal* food is reviewed based on the papers cited above [1,2,3,4,5,6,7,8,9,10,11,12,13,14,15,16,17,18,19,20,21,22,23,24,25,26,27,28,29,30,31,32,33,34,35,36,37,38,39,40,41,42,43,44,45,46,47,48] with special discussion given to *Halal* certification.

One of the earliest requests for *Halal* assurance and certification in Malaysia was made to Muis in 1978 concerning the veracity of *Halal* labelling by food producers and the misuse of *Halal* claims on a noodle product containing pork [121]. In 2005, Ingvar Kamprad Elmtaryd Agunnaryd (IKEA; a Swedish multinational conglomerate) Malaysia faced one of their most serious challenges since they began operating in the country when their restaurants were raided by government officials on suspicion that food served there did not comply with strict religious dietary regulations in the country [41]. According to Arif et al. [39], the Department of Islamic Development Malaysia (JAKIM) has used the halalan tayyiban concept to determine what constitutes *Halal* cuisine in Malaysia. These results contribute to consumers having more faith in the food that JAKIM has deemed to be *Halal*. They have also helped grow the well-being and sustainability of communities, especially the Muslim community. According to Ibrahim et al. [23], organic certification is one kind of food certification used to demonstrate product attributes to win consumer trust and distinguish between organic and non-organic products.

The idea of *Halal* food items is gaining popularity because it is a substitute standard for ensuring the quality, safety, and hygienic conditions of what can be ingested. Hence, products or foods produced in line with *Halal* prescriptions are acceptable to Muslim consumers [138]. The issue of *Halal* cuisine has inspired a continually expanding interest, especially in recent years, and it is one of the most prominent topics on the agenda of both the scientific and corporate sectors [42]. *Halal* cuisine is well known for its outstanding wholesomeness and quality guarantees, which draws both Muslim and non-Muslim customers [30]. *Halal* food is food approved under Islamic law. On the contrary, haram food is not authorised, such as alcohol, pork, blood, carrion, and meat not killed according to sharia [7]. Islamic jurisprudence’s rules for what can and cannot be consumed have been used to evaluate *Halal* food [126]. *Halal* food is defined as being morally right and legal. No matter where a Muslim lives or travels, eating *Halal* food is a religious necessity for everyone who practises their religion [120]. *Halal* foods must never include alcohol for any purpose; yet, commercial rice cakes sometimes contain ethanol as a preservative [94]. The parameters for *Halal* food practises have been determined according to the sources of ingredients, the methods of slaughter and the handling, distribution, storage, and presentation of products along with hygienic and legal standards [143]. Muslims must make the best decisions possible in all areas of life, particularly when dining [127].

The construction of what is considered to fall within the *Halal* parameters set by the Quran and Sunnah—the two main sources of sharia—and the issuance of fatwas by national religious authorities depends on the process of *Halal* certification [35]. Based on all of the aforementioned research, the *Halal* certification of biofertilizer for food production thus presents a substantial potential for future debates from a socio-economic point of view.

### 1.2. The Concept of Effective Microorganisms

To boost the microbial diversity of soils and plants, effective microorganisms (EMs) are mixed cultures of advantageous and naturally occurring microorganisms that can be used as inoculants [145]. They add a new layer to optimizing our best soil and crop management strategies such as crop rotation, using organic fertilizers, conservation tillage, crop residue recycling, and pest biocontrol. If appropriately applied, these advantageous microorganisms can greatly increase the positive results of these techniques [146].

EMs are a mixture of advantageous, naturally occurring microorganisms that can be used as inoculants to boost the microbial diversity of a soil ecosystem. An EM can be found as a liquid concentrate and is made using a natural fermentation process; it is not chemically or genetically altered. Various advantageous, naturally occurring microbes, primarily found in or used for food, are combined to form an EM. Over 80 different types of microorganisms are grown in vats to generate them [145,146].

**Figure 1:**
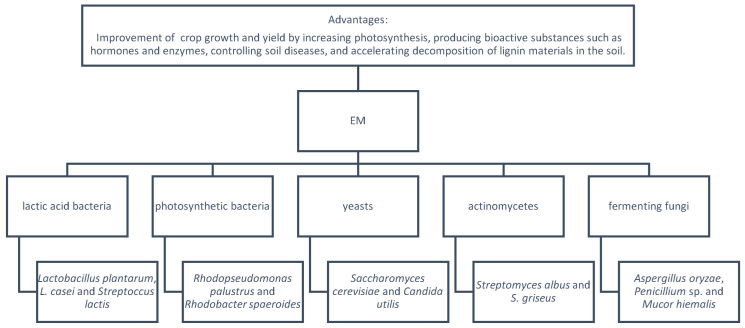
Effective microorganisms (EM) with their compositions [147,148,149].

Figure 1 lists the components of EMs. An EM is composed of 10 genera from 5 families, including photosynthetic bacteria (*Rhodopseudomonas palustrus* and *Rhodobacter spaeroides*), lactic acid bacteria (*Lactobacillus plantarum*, *L. casei*, and *Streptoccus lactis*), yeasts (*Saccharomyces cerevisiae* and *Candida utilis*), actinomycetes (Streptomyces), and fermenting fungi [147]. However, the main components of EM are lactic acid bacteria, yeasts, and photosynthetic bacteria [148]. In liquid culture, all of these can coexist and are mutually compatible [149].

The bacteria that use photosynthesis are autonomous, self-sustaining creatures. The exudates from root systems, soils’ organic fractions, and gases such as ammonia are converted into cell components using energy harvested from the sun and soil heat. Some of these can be absorbed directly by plants, promoting plant development and supporting other EMs’ growth and establishment in the soil system. Amino acids, polysaccharides, nucleic acids, bioactive compounds, and sugars are among the valuable molecules created by photosynthetic bacteria; all of which aid in the growth and development of plants [150].

Lactic acid bacteria use sugars and other carbohydrates from organic matter, including photosynthetic bacteria and yeasts, to produce lactic acid [151]. Strongly sterilising lactic acid also suppresses dangerous germs such as *Fusarium* and hastens the degradation of organic materials [152]. It is known that photosynthetic bacteria and yeasts in EMs produce sugars and carbohydrates, which lactic acid bacteria in EMs use to make lactic acid. Because of its sterilising properties, lactic acid inhibits nematode population growth and provides protection against plant illnesses brought on by nematodes in the soil. Additionally, the soil’s digestion of lignified and cellulolytic organic compounds is aided by lactic acid bacteria found in EMs [153].

Contrarily, the yeasts in EMs release hormones and enzymes that are known to promote the division of plant cells and roots. Utilizing the sugars and amino acids released by plant roots and photosynthetic bacteria, they create growth factors for lactic acid bacteria. From amino acids and sugars released by photosynthetic bacteria, organic matter, and plant roots, yeasts make beneficial chemicals necessary for plant growth [150]. Yeast produces bioactive compounds that encourage vigorous cell and root division, including hormones and enzymes [154].

All of the aforementioned information shows that many EMs work in harmony with one another and have a mutually beneficial relationship with the roots of plants. Therefore, plants thrive particularly well in soils where these EMs are present and dominant.

### 1.3. Beneficial Effects and Advantages of Effective Microorganisms in Agricultural Production

The key advantage that an EM has over natural organisms in organic amendments is that when EMs are introduced, they are substantially more numerous and in optimally balanced populations. As a result, they stay in the soil environment for a considerably longer period of time: long enough to have the desired effects.

According to studies, using EMs in agricultural soil reduces the presence of soil-borne pathogens and hastens the breakdown of organic matter, increasing the amount of mineral nutrients and essential organic compounds available to plants [155]. The fight for resources between disease-causing microorganisms in the soil and beneficial bacteria added through Ems results in disease suppression. Because of this, an increased population of EMs from inoculation exhausts the soil’s nutrients, which causes harmful bacteria to starve to death [156].

Additionally, EMs promote the growth of helpful native microorganisms, such as mycorrhizae, which fix atmospheric nitrogen in place of commercial fertilizers and pesticides. Crop plant growth, flowering, fruit development, and ripening are all significantly and positively impacted by improvements in soil fertility [157]. There is proof that EM inoculation of soil can enhance soil quality, plant growth, and crop yield [158]. These EMs increase photosynthesis, produce bioactive chemicals such as hormones and enzymes, suppress soil infections, and hasten the breakdown of lignin components in the soil, increasing crop development and yield [151]. In general, applying EMs enhances the physical and chemical characteristics of soil and promotes the development and effectiveness of symbiotic microorganisms such as nitrogen-fixing rhizobia and arbuscular mycorrhizal fungi [159]. These EMs can coexist in liquid culture since they are physiologically compatible with one another.

Any soil system with these EMs can break down and ferment the organic portion, turning it into a humus containing nutrients and releasing hormones that promote plant growth. Through the root system, these microorganisms provide plants with hormones, nutrients, and minerals in a form they can use. Additionally, they assemble soil fragments in the soil structure, allowing it to retain nutrients and moisture [158]. Agriculture production is significantly influenced by soil conditions, reflecting a complex web of biological, chemical, and physical interactions fuelled by microorganisms. Effective microorganisms boost the soil’s EM population, which is necessary for crop productivity over time. Ems, which may be utilized as inoculants, are mixed cultures of advantageous, naturally occurring organisms that can increase the soil environment’s microbial diversity. They generally consist of lactic acid bacteria, yeasts, actinomycetes, bacteria that can create light, and fermenting fungi. EMs improve the structure of the soil, increase its fertility, dramatically increase biological diversity, suppress soil-borne pathogens, improve soil quality, increase the beneficial minerals in organic compounds, and increase plant vigour and crop yield. EMs work by eliminating other soil bacteria. As a result, most other soil microorganisms begin to follow them, inhibiting the activity of the smaller number of unfavourable or opportunistic bacteria. Effective microbes can help improve and preserve the chemical and physical properties of soil [160].

In summary, the beneficial effects and advantages of effective microorganisms have been well documented in the literature, ranging from various crops to cash-commodity oil palm crops [161,162,163].

### 1.4. Why Focus on Halal-Based Sources for Biofertilizer Production?

To create food, fibre, medicinal herbs, and other things for human use or, more specifically, to support and enhance life, agriculture is the activity of cultivating land and raising animals and plants [17]. Research on *Halal* food has significantly increased during the last 10 years [3,17,99,164]. The food business has long been regarded as a successful sector because of its contribution to the national economy, the generation of jobs, and the welfare of people [165]. *Halal* food supply chains are growing more disjointed, convoluted, and unpredictable. *Halal* food consumers are becoming increasingly discriminating regarding food quality and adherence to Islamic dietary laws [166]. Despite sustainability being a significant concern in supply chain management, sustainability research in *Halal* food supply chains is still in its infancy [167]. Additionally, the advancement of the *Halal* certification and the blockchain tool could enhance sustainable development through fair trade, ethical business, green animal breeding, and environmental economics, even though this will require significant innovation and collaboration among the local government, business associations, and influential consumers [59]. According to Samsudin and Dani [168], there is a rising trend in the number of publications on research regarding *Halal* manufacturing, with the three major themes being adoption of *Halal* certification systems, improvement of *Halal* manufacturing processes, and *Halal* operational capabilities.

It is well known that microorganisms may be distinguished from various settings, including leaves, fruits, soil, water, and milk. The employment of microorganisms in fermentations that are obtained from a *Halal* and hygienic environment is essential for generating a final *Halal* product. According to research, some probiotic bacteria, antimicrobial reuterin, and bacteria that produce non-steroidal estrogen and are isolated from pigs should not be utilized to produce *Halal* microorganisms. Non-*Halal* fermentations include those that use microorganisms that have been isolated from non-*Halal*-based sources such as blood, animal faeces, dead animal body parts, etc. [168,169].

Nonetheless, several studies in the literature have used microorganisms of a haram origin, such as pork-related faeces. In several studies, to produce proteases, amylases, pectinase, xylanase, cellulase, lipase, and ligninase for commercial application, several microorganisms were isolated from a variety of settings, including dead animals, animal faecal deposits, freshwater ice, soil, silt, algae, plants, and rocks [170]. Several lactic acid bacteria (LAB) strains were also discovered in human faeces, and their usage in creating fermented meals made of various vegetables was investigated [171]. Using spray- and freeze-drying dehydration methods, Zamora et al. [172] investigated the long-term preservation of 12 LAB cultures derived from pig blood. There does not seem to be any research demonstrating whether bacteria of impure origins may eventually become *Halal* in media based on *Halal* principles.

This paper aims to review the literature on ‘Effective Microorganism’ and ‘fertilizer’ from the Scopus database and to discuss EMs using *Halal*-based sources for biofertilizer production from a socio-economic perspective.

## 2. Methodology

**Figure 2:**
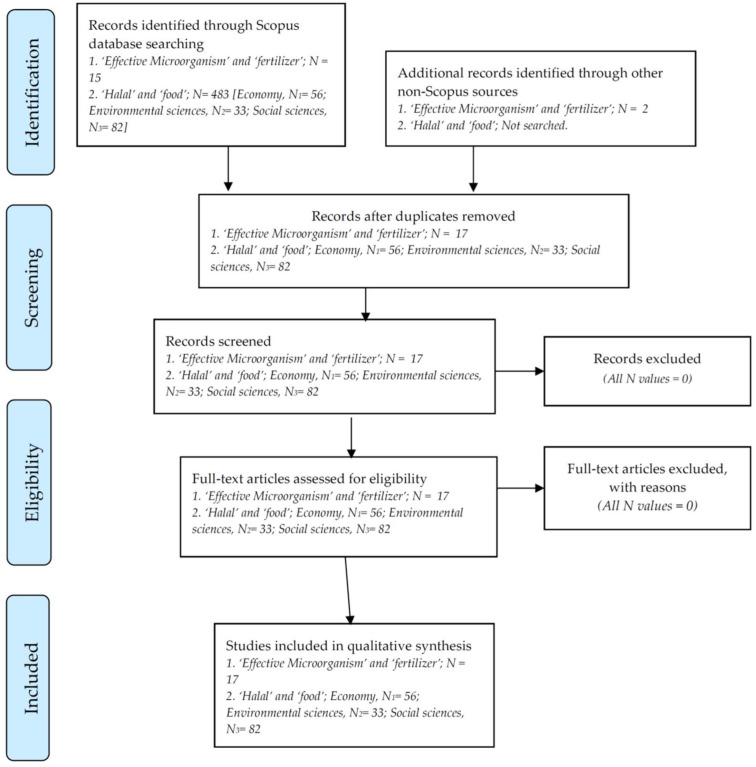
A flowchart of Preferred Reporting Items for Systematic Reviews and Meta-Analyses (PRISMA) that was used in the present study adapted from Moher et al. [173].

The systematic literature review methodology of Preferred Reporting Items for Systematic Reviews and Meta-Analyses (PRISMA) by Moher et al. [173] was used in the present review study. PRISMA is an evidence-based reporting standard that is effective for critical appraisal. Overall, the steps of systematic methodology adapted for this review article are shown in Figure 2. The database Scopus was used for literature analysis because it is the most common database for conducting literature searches.

Based on a search using the Scopus database on 29 February 2023, there was no publication title that included the keywords ‘*Halal*’ and ‘fertilizer’. The absence of these two keywords on the topics of the publications searched in this paper indicates that studies on this topic are still in their infancy at most. However, when the keywords ‘Effective Microorganism’ and ‘fertilizer’ were combined, 15 publications with these keywords in their titles were discovered.

In addition, from a search using Google (non-Scopus), 2 papers related to ‘Effective Microorganism’ and ‘fertilizer (or fertiliser)’ were also included since they are relevant to the present topic of discussion.

To understand the above articles containing the keywords ‘Effective Microorganism’ and ‘fertilizer’, a discussion based on two major perspectives, society and the economy, must be had. To do this, another review was performed. A search based on keywords ‘*Halal*’ and ‘Food’ contained in article titles using the Scopus database was performed on 17 March 2023. There was a total of 483 papers recorded. Of this total, 56 papers were categorized under ‘economics, econometrics and finance’ in the Scopus database. Of these, there were 33 articles categorized as ‘environmental sciences’ in the Scopus database (Figure 1 and Table S1). Out of the 483 papers, 82 were categorized as ‘social sciences’ in the Scopus database. Interestingly, the largest number of papers related to ‘social sciences’ were connected to ‘economics, econometrics and finance’ (14 papers [17%]; socio-economy) and ‘environmental sciences’ (9 papers [11%]; socio-environment) (Figure 2).

## 3. Results

The detailed review for each publication is discussed in Table 1.

With the inclusion of water hyacinth and EM4, Ali et al. [174] investigated industrial rubber waste’s use in producing liquid compost fertilizer. Bzdyk et al. [177] examined the effects of (a) EM composed of mixed cultures of helpful and naturally occurring microorganisms, (b) the organic fertilizer Actifos, and (c) the effect of the mineral fertilizer Busz Forte on the growth and mycorrhizal colonization of two common deciduous forest tree species after one growing season in a bare-root forest nursery (*Fagus sylvatica* and *Quercus robur*). In contrast to nitrogen and phosphorus fertilizers, Chantal et al. [175] studied the effects of EMs on the leaf areas and photosynthesis behaviours of vegetable cabbage. They discovered that EMs increased plant output and cabbage quality.

The growth characteristics and yield quality of a flue-cured tobacco plant treated with EMs and two different amounts of bio-organic fertilizers were later evaluated by Chantal et al. [176] using a lysimeter experiment. They concluded that EMs used in conjunction with bio-organic fertilizer successfully raised the tobacco plant’s leaf chlorophyll content. *Aspergillus niger*, *Pseudomonas putida*, and EM4 were used in a study by Darmawan et al. [179] to examine the potential of coconut water as a liquid organic fertilizer. The results employing EMs were promising. Elpawati [180] sought to develop an EM-10 e-commerce information system. An organic waste activator called EM-10 can hasten the decomposition of organic waste and create odourless trash. Their research aimed to create an e-commerce system for an online store to present EM-10 and arrange customer, product, and trading data.

Iwaishi [181] investigated how 13 paddy-rice types with varied maturity times responded to the growth, yield, and quality effects of an organic fertilizer inoculated with EMs. The glutinousness and overall quality index of sticky rice cultivars were both improved through EM inoculation. Early and medium-ripening non-glutinous types and glutinous varieties were suitable for natural farming with EM-inoculated organic fertilizer under the climatic conditions of 1993. Javaid [182] examined the effects of foliar and soil applications of these microbes on pea (*Pisum sativum* L.) growth, yield, and nodulation in soils treated with green manure from *Trifolium alexandrinum* L., farmyard manure and NPK fertilizers. The findings demonstrate that foliar EM treatment increased nodulation using NPK amendment, with the nodule number increasing by 217% and nodule biomass increasing by 167%. These results demonstrate that foliar EM spray and the right soil amendment can increase pea nodulation and production.

Ncube et al. [183] conducted a greenhouse experiment to evaluate Swiss chard’s (*Beta vulgaris* subsp. cicla) sensitivity to EMs and the chemical characteristics of the soil. However, their findings supported the fact that the use of EMs produced variable effects on Swiss chard yields and soil characteristics. The impact of EMs combined with liquid organic fertilizer on the development and quality of Pak Choi seedlings was examined by Riddech et al. [184]. Sulistyaningsih et al. [178] sought to process the vinasse of the alcohol industry as an efficient growing medium of environmentally friendly microorganisms that can be used in producing organic fertilizers. They discovered that organic fertilizers with EMs benefited Pak Choi through the reduction of leaf senescence when compared to other treatments. Their study of EM culture showed an increase in the number of microbes. Additionally, their obtained organic fertilizer contained amounts of nitrogen, phosphate, potassium, and organic matter totalling 43.4%, 3.05%, 0.40%, and 1.23%, respectively. A study by Yamada and Xu [158] sought to clarify the chemical, physical, and microbiological characteristics of an organic fertilizer that had been infected and fermented with an EM. The organic fraction, direct impacts of the introduced microorganisms, and indirect effects of metabolites produced by the microbial process likely determine the beneficial benefits of the fermented organic fertilizer on soil fertility and crop growth.

According to Li et al. [185], applying an efficient microbial biochar-based fertilizer was positively connected with the net photosynthetic rate, stomatal conductance, intercellular CO_2_ concentration, and transpiration rate at the development and maturity phases of tobacco. The yield of tobacco (*Nicotiana tabacum* L.) was favourably connected with the quantity of irrigation and EM biochar-based fertilizer, according to Li et al. [186].

In a two-year field experiment to investigate how stevia plants respond to nitrogenous fertilizers (NFs) and environmental pollutants (EPs), Youssef et al. [187] investigated the impact of various fertilizers and biofertilizers on stevia plants. Overall, applying NFs and EPs together boosted stevia plant growth, yield, and nutrient uptake.

Because of this, Abdullah et al. [188] advised adopting a *Halal*-based source for applying EM technology in Malaysia, which is not limited to agriculture. Sulaiman et al. [189] found that the nutrients in compost-based fertilizer plants in Brunei, were all in the acceptable range. it exemplifies how composting home food waste may support Brunei’s efforts to manage waste reduction for a sustainable and healthy environment as well as a circular economy. Although fascinating, their research does not address the topic of EMs in agriculture.

## 4. Discussion

### 4.1. Lack of Information on Effective Microorganisms Using Halal-Based Sources for Biofertilizer Production

Based on EM and fertilizer publications obtained from the Scopus database, all 17 papers reviewed above provided no detailed information on the *Halal* status of organic and bio-organic biofertilizers inoculated with EMs. Hence, this review shows that studies on the *Halal* statuses of fertilizers (or biofertilizers) using EMs are greatly needed in future studies. This knowledge gap is interesting for future studies.

The use of EM solutions in manufacturing biofertilizers also aids in the growth of EMs in the soil, which enhances the soil’s microbial health and fosters a favourable environment for plant growth [190]. Particularly, there is uncertainty regarding the *Halal* status of the sources of EM. Therefore, these ambiguous circumstances could lead to *Halal*-status concerns among customers who are primarily Muslims. Therefore, it is advised to produce EMs from *Halal*-based sources (for instance, fruits) rather than importing EM stock from nations with non-Muslim dominance. Initially, EMs were only available dormant and needed to be activated before use. To activate latent EMs, water and jaggery were added [191]. According to a prior study, since EM liquid concentrate’s raw components are produced by several companies, they are frequently not explicitly mentioned or identified. Particularly, there is uncertainty regarding the *Halal* status of the sources of EMs. According to this perspective, most Muslim consumers may have issues about whether certain circumstances are *Halal*.

Numerous applications of EMs have been demonstrated, including those in farming, raising cattle, gardening, landscaping, composting, bioremediation, cleaning septic tanks, controlling algae, and domestic uses [149]. Appropriate formulation processes are essential for the effective usage of EMs. Ems perform better if they are combined with appropriate nutrients, adhesives, or wetting agents [191]. As a result, scientists are very interested in the potential applications of organic fertilizers (such as animal dung) and inoculants of advantageous microorganisms in advancing agriculture. The potential for treating pig dung with EMs before feeding it to fish was raised by Hanekom et al. [192]. Thus, the *Halal* status of such a product is primarily a concern for Muslim consumers who are restricted from handling pig excrement. Muslim consumers are becoming more concerned about products’ *Halal* statuses today.

According to Chaudry and Regenstein [193], genetic alterations that enhance product flavour, colour, texture, and composition may not raise any issues with Muslim acceptance if such items are metabolized by the human body in the same way and are otherwise safe to consume. In this situation, questions about the *Halal* status of genetically modified organisms such as EMs have been raised. The *Halal* implications of EM production have not yet been studied in any depth. Research on certain agri-food items shows great potential because countries such as Malaysia and Indonesia depend heavily on agriculture. The agriculture sector is Malaysia’s third development engine, and the agricultural biotechnology sector shows significant local growth potential. Frost and Sullivan [194] mentioned a potential global *Halal* supplier (GHS) as one of Malaysia’s four primary important prospects for agriculture biotechnology. The *Halal* industry is a rapidly growing business and has the potential to become a GHS. Rising wages and consumption in important Muslim markets, as well as the consistently rising annual sales of *Halal* food products, are supporting the continued expansion of the country’s economy.

The Organization of Islamic Conference (OIC) also appreciates Malaysia’s leadership in *Halal* concerns. The Department of Islamic Development Malaysia (JAKIM), the designated *Halal* certification organisation, uses the *Halal* standard for its *Halal* Certification programme. The guideline places emphasis on where *Halal* food comes from, which can include both land and water animals as well as plants, mushrooms, microbes, natural minerals, chemicals, and beverages [194]. To access the plantation markets of Islamic countries, the EMs utilised in biofertilizers must come from *Halal*-based sources.

Malaysian agricultural products are positioned to serve both the local and world markets by capitalising on the country’s potential to become a GHS. *Halal* cuisine is prepared according to a set of Islamic dietary laws and guidelines that specify what is acceptable, legal, and pure. Food should be prepared from crops and plants fertilized with *Halal*-based biofertilizers. It is crucial that Malaysian *Halal* goods reach the international market. As a result, even though this biotechnology may encounter some difficulties, the biofertilizers produced via EMs should come from *Halal*-based sources because they are regarded as novel tools for agriculture [190,195].

### 4.2. The Potential Impacts of Halal-Certified Biofertilizer from a Socio-Economical Perspective

*Halal*-certified biofertilizers are eligible for *Halal* certificates: a necessary and subsequently trust-generating requirement for consumers of many *Halal* food items. As indicated in Table 2, many positive socio-economic implications follow from *Halal*-certified biofertilizers in agricultural food products. This can be judged from the following relevant literature.

(a) *Cater to the increasing Halal food demand due to expected Muslim population expansion*

There are now more Muslims globally than ever before. As of 20 March 2023, it is anticipated that 25% (>2.01 billion) of the world’s population identify as Muslims, making Islam the second most practised religion in the world [196]. Since Muslims currently make up more than 25% of the world’s population, and the number of Muslim tourists has significantly expanded in recent years, it is vital to investigate the potential benefits of *Halal* cuisine for the tourism industry and worldwide trade [141]. The market for *Halal* food is progressively growing [94]. The demand for goods and beverages with the *Halal* label is increasing as a result. Food quality is also among the most well-known features of the *Halal* certification and logo, and it appears to be accepted as a key factor in making restaurant patrons happy [134].

Future demand for *Halal* food is predicted to rise as the number of Muslims worldwide rises. The demand for *Halal* products is therefore anticipated to expand rapidly, making it essential to improve the traceability and dependability of its worldwide market. More *Halal*-certified goods should be available for household consumption in nations with mostly Muslim populations [57]. Due to demands from the OIC to fulfil *Halal* product guarantees and food safety, Indonesia, the nation with the largest Muslim population, may become the largest exporter of *Halal* food goods globally [58]. In Indonesia, young people possess a high level of consumer knowledge regarding *Halal* cuisine according to Khaliqi and Pane [62]. Companies possessing *Halal* certifications show a stronger propensity for corporate social responsibility, according to Secinaro et al. [67]. Thus, a *Halal*-certified biofertilizer could be a wise choice.

During the past ten years, *Halal* food research has seen a noticeable increase [3]. As Muslim communities grow, so too does the demand for products and services that comply with *Halal* standards [5]. Creating *Halal* versions of food products could be the answer to meet this demand. Ariffin and Fadzlullah [111] demonstrated that certain local producers did not exhibit any certificates for their food goods despite significant requests for *Halal* certification. Malaysia is advancing towards becoming a centre for *Halal* manufacturing and certification worldwide.

*Halal* products are quickly gaining popularity worldwide because there are more Muslims, and more importantly, because *Halal* is now seen as a new standard for quality assurance and safety. *Halal*-certified biofertilizer producers must comprehend *Halal*-based supplies and related production procedures to satisfy consumer expectations. To introduce the idea of *Halal* in EM technology and to sustain agriculture output for the socio-environmental preservation of ever-increasing human populations, the idea of developing EMs using *Halal*-based sources for biofertilizer production is highly intriguing. It is almost a given that sustainable agriculture, including palm oil production, will boost the nation’s economy.

When Muslims and people from other cultures purchase *Halal* food, their quality of life rises [99]. The quantity of geographical studies in the literature examining food/animal ethics that highlight the expanding importance of the *Halal* food business is rising [104]. In Malaysia, Muslim consumers commonly express reservations about the concept of a *Halal* food supply chain [110]. However, the increased concern for health presents a huge opportunity for the *Halal* food business to draw non-Muslim consumers. Masrom [114] provided novel viewpoints on business excellence, especially with respect to *Halal* production. To accommodate Muslim travellers’ dietary requirements, particularly in non-Muslim countries, it was found that some of them had already started promoting *Halal* food.

(b) *Contribute to the sustainable buying behaviour of consumer of Halal products in the future*

A preliminary study by Kurokawa [88] established that the *Halal* food logo provides a community with new branding value. Customers are becoming more selective about the food they buy due to growing knowledge of *Halal* food products. The assumption made by marketers is frequently that customers would buy food goods if *Halal* markers are labelled [19]. Customers of a large retail hypermarket chain in Malaysia participated in a study that was conducted by Hamzah et al. [19]. Retailers may use these results to ensure that their food goods possess these components in order to entice Muslim customers to buy them. Wiyono et al. [57] conducted an in-depth analysis of Muslim consumers’ motivations for purchasing *Halal* food products. This study can help analyse intention factors while studying *Halal* food products. *Halal* certification significantly improves the marketability of products sold by SMEs according to Anggarkasih and Resma [58]. The government’s tangible actions in putting the *Halal* certification for SMEs into effect might be the deciding factor. This demonstrates the significance of *Halal* certification of processed goods to improve export potential. In the context of Malaysia’s multicultural culture, Hassan et al. [38] examined the usage and efficacy of customers’ perceptions of *Halal* certification as a quality assurance mark.

The opinions of Thai Small and Medium Enterprises business owners towards *Halal* Food Certification were examined by Abdul et al. [47] concerning market share and market competitiveness, government assistance and oversight, and information sharing about *Halal* hubs. Their survey also revealed that respondents’ marital status and gender greatly impacted whether they planned to apply for a *Halal* certificate or not as well as whether they wanted to obtain *Halal* certification for their food items. To increase customers’ trust in these goods, Rezai et al. [51] suggested that frequent monitoring and enforcement of *Halal* rules and regulations be carried out. Mostafa et al. [20] state that consumers of *Halal* cuisine appear to be a very diverse community, with differences in their level of religion, sense of self, views towards animal welfare, and concern for dietary authenticity. Azizah [99] stated that Indonesia needs legislation to regulate *Halal* food because the Muslim community exclusively eats *Halal* food. Damit et al. [30] revealed a significant relationship between the attitude of non-Muslim consumer towards *Halal* products and their repurchase intentions of *Halal* products.

Muslims, especially Generation Z members, who prefer eating in stalls and restaurants rather than at home, must prioritise *Halal* food production [1]. According to earlier research conducted in Muslim-majority nations such as Saudi Arabia, Islamic brands do not increase interest in purchasing *Halal* food. This finding was confirmed by Febriandika et al. [1]. This is so they can continue to purchase *Halal* food without seeing the Islamic brand. However, this result may differ if Muslims live in a non-Islamic country since Islamic branding can provide a comfortable feeling for Muslims in non-Islamic countries.

According to Bawono et al. [8], purchase intention, enabling circumstances, and habit directly impact whether or not someone buys *Halal* food. With their acceptance of and feelings towards Korea’s image and its *Halal*-food image, Yang et al. [11] considered the differences between Indonesian and Malaysian consumers. These conclusions regarding Korea’s reputation may be crucial in formulating the country’s food export strategy and may have strategic ramifications for Korean businesses seeking to enter the *Halal* food market or grow their market shares. According to Astuti et al. [13], *Halal* awareness plays an important role for Muslims in the decision-making process for purchasing food. Contrary to the initial idea, the nation of origin really did not have a favourable influence on sentiments regarding the *Halal* label. Sudarsini and Nugrohowati [16] indicated that the relationship among religiosity, knowledge, and attitudes positively influenced consumer intention to consume *Halal* food, cosmetics, and pharmaceutical products in Indonesia.

(c) *Cater to the increasing number of Muslim travellers around the world*

For managers and service providers in the hotel sector, the study results of Mannaa [105] help reveal the significance of *Halal* meals for Muslim travellers. The findings also allow the Destination Management Organizations to better market and provide products to Muslim visitors and raise the understanding of non-Muslims regarding *Halal* principles and associated culinary items. Yazawa and Kikuoka [100] discovered that information regarding *Halal* meals was important, and with the rising number of Muslim travellers to Japan, it appears vital to actively develop opportunities to learn about *Halal* foods through registered dietitian training courses in the future. To ensure the safety and *Halal* status of ready-to-eat local food and to support the development of culinary tourism in Surabaya, Indonesia, there needs to be a greater awareness of and practicality concerning tracing ingredients, production, and serving practises [63].

To increase Muslim tourists’ trust in the nation’s food products, *Halal* product guarantees must be properly implemented according to Nuraini and Sucipto [69]. The implementation of standards and regulations in each country is different. Each nation creates *Halal* and high-quality goods in tourist areas for Muslim travellers worldwide, increasing the number of trips. *Halal* tourism has become one of the crucial industries to boost the economy of the country and the people’s income in the country. A significant portion of companies sampled in a survey conducted to study how *Halal* food is managed and promoted in New Zealand restaurants by Wan Hassana et al. [53] did not agree that the Muslim tourist market was important to their company. Several were also unwilling to market their *Halal* meals or put up ‘*Halal*’ signs in front of their establishments [33]. A *Halal* culinary tracking application has been created by Sucipto et al. [32] to make it easier to find *Halal*-certified souvenirs in Malang City, Indonesia.

Indonesia, which is rich in tourism and gastronomy, shows huge potential to grow *Halal* tourism. The development of *Halal* restaurants and *Halal* food souvenirs in *Halal* tourism in Indonesia has to be centred on food quality, service quality, and environmental quality according to *Halal* foods sensory appeal, usefulness, and symbolism [77]. According to Perguna et al. [101], *Halal* kiosks in Denpasar Bali, Indonesia, aid in tourism by offering amenities and services. This helps the area’s reputation as a tourist destination. Sales and consumption of *Halal* items have significantly grown since *Halal* cuisine was introduced in the US [102]. To determine the extent of *Halal* food tourism’s contribution to the economy of the city’s tourism sector in Durban, South Africa, Bhoola [106] advises a large-scale quantitative study. It may be possible for the *Halal* food industry to grow internationally as a result of the rising demand for *Halal* food among Muslims who visit or live in non-Muslim countries [115].

An increase in national income is the direct result of the above-mentioned rise in Muslim travellers worldwide due to the impact of *Halal*-certified biofertilizer. Eventually, this can result in nation-building owing to economic progress. Increase the cost-effectiveness and marketability of food products, thus positively impacting national income. The following literature highlighted the importance of the economic perspective on *Halal* food, which triggers *Halal* certification for industrial biofertilizer products.

The concept of *Halal* products or food is gaining acceptance worldwide as a new norm for assuring food security, sanitary conditions, and quality of what is consumed. As a result, many non-Muslim consumers, as well as Muslims, will accept products or meals that have been prepared in line with *Halal* laws [138]. Given the exponential expansion of the international *Halal* food market, Malaysia now faces a great opportunity to develop itself in this area [110,111]. Idris et al. [50] discovered in 2013 that Malaysian *Halal* food companies were still unprepared to enter the global market by establishing strong business networks, particularly marketing networks. To establish and maintain a competitive edge in the worldwide market, Camillo et al. [43] studied marketing techniques utilized to internationalise the *Halal* food sector. To ensure that a system is put in place that can meet their needs in a way that is both affordable and provides a truly *Halal* and tayyab (wholesome) food supply for Muslims in all countries, whether Muslim-majority or not, the Muslim community will need to collaborate with the food industry and Muslim certifying agencies according to Regenstein et al. [45]. Nursalwani et al. [69] revealed that young entrepreneurs demonstrate high engagement levels, and they also revealed a strong association between societal norms and perceived behaviour towards involvement in *Halal* food product labelling in Kelantan, Malaysia. According to Ali and Suleiman [36], compliance with environmental requirements can go hand in hand with fulfilling other sets of manufacturing standards.

Government support infrastructure is necessary to sustain the industry’s development. For the Philippine Food *Halal* Industry to thrive in both local and export markets, technological research and development with respect to quality assurance and traceability are required [66]. According to Wiryani et al. [81], reforming criminal punishments, enhancing professionalism and sensitivity of law enforcement, and augmentation of community involvement are necessary for optimising law enforcement of *Halal* and thoyib food consumer protection. Due to the increased number of customers and the worldwide market of *Halal* products, several research studies have concentrated on *Halal* food supply chains [86]. Masrom [114] presented fresh insights into the domain of business excellence, specifically connected to *Halal* manufacturing in Malaysia.

A growing industry in Thailand is the certification of food as *Halal* for export to Muslim nations such as Malaysia and Indonesia [88]. According to Widyantoro et al. [115], the necessity to satisfy Muslims’ eating requirements overseas, notably in non-Muslim nations, has led certain governments to promote *Halal* cuisine. The growing demand for *Halal* cuisine among Muslims travelling to or residing in non-Muslim nations may present an opportunity for the *Halal* food sector to expand globally. The effect of *Halal* certification on Indonesian restaurant business performance was examined by Yama et al. [116]. The researchers discovered that *Halal*-certified restaurants emphasise upholding moral standards and legal requirements to maximise production. This has enormous consequences for the Indonesian restaurant business since *Halal* certification may enhance performance. Yener et al. [42] identified opportunities for the logistics industry as well as the potential of the global *Halal* food market.

The growing *Halal* food economy’s effects on the American food industry and its proportion of the expanding global *Halal* food economy were examined by Halawa [120]. There is a need for greater research to explore the long-term socio-economic and environmental sustainability impact on expanding international Muslim communities living in low-income countries. Ariffin et al. [12] proved that utilitarian and knowledge functions directly impacted the opinions of these Muslim communities regarding *Halal* food operators in which non-Muslims operate. This may aid non-Muslim owners of *Halal* food establishments in creating the best plans for capturing Muslim consumers’ hearts and resulting in increased business.

Arieftiara et al. [5] concluded that *Halal* certification positively impacts corporate performance because it signifies a commitment by food and beverage companies to provide products and services according to sharia law. Research has suggested that converting to *Halal* products and services might increase the success of a business. Hamid et al. [6] revealed that the economic scale of trading partners, regional trade agreements, shared borders, and shared language greatly favourably affect the value of *Halal* food and beverage exports. As a Malaysian *Halal* food standard, MS1500 was designed to boost Malaysian involvement in the worldwide *Halal* market [10]. According to Perdana et al. [21], the Middle Eastern and North African (MENA) region’s food business operators would benefit from the existence of *Halal* certification. Despite being Muslim-majority countries, it is important to ensure the presence of *Halal* certification in MENA countries’ products, especially those that received low scores in the country-of-origin study.

In the *Halal* supply chain, protecting the products’ *Halal* integrity has evolved into a component of ethical business practice. From farm to fork, the *Halal* supply chain should be entirely healthy [18]. Parlak [22] claims that the idea of *Halal* food will be regarded as a religious obligation and that the idea of “*Halal* food” has achieved a foothold in the market. They illustrated the political and economic incentives behind *Halal* food, in conjunction with developments in religion, with Turkey as the major emphasis.

The result of the aforementioned cost-effectiveness and rising marketability of food brought on by the use of a biofertilizer with a *Halal* certification is an increase in national income. This would subsequently lead to the nation’s socio-economic development.

(d) *A positive driver for higher production of more Halal foods that can enhance food safety, human health, and well-being, thus increasing national income.*

To ensure that food is free of any ingredients that are forbidden by Islamic law, *Halal* authentication has become crucial in the food industry. Muslim consumers are concerned about diversifying food origins and adulteration issues [60]. Verification of food ingredients and their purities are therefore crucial. Due to customers’ and producers’ disregard for the significance of safety and *Halal* status in their products, the danger of safety and *Halal* status in local meals is fairly high. To ensure the safety and *Halal* status of a product, an area’s speciality foods should actually receive more attention [63]. Muslims all over the globe place great importance on the *Halal* food sector because it ensures the security and safety of their consumption and nutritional needs, both of which must comply with sharia [43].

The limitations and difficulties that businesses face when implementing the *Halal* Assurance System in an integrated management system were covered by Puspaningtyas and Sucipto [71], who also discussed how these challenges have an impact on a company’s performance in terms of productivity, quality, safety, finances, and risk reduction. Only two factors—the *Halal* logo and product quality in Indonesia—have a major impact on consumers’ intentions to buy *Halal* food according to Arifin et al. [73]. Kurokawa’s early research [88] confirmed that the *Halal* food industry is growing in non-Muslim nations. Preliminary research by Kurokawa [88] confirmed that *Halal* certification offers a good opportunity to improve security of the food trade. Yousaf [90] claimed that Muslim visitors consider *Halal* food a very significant resource. Yet, the socio-spatial confinement of quarantine restricts the supply of true *Halal* food, causing *Halal* food anxiety to take hold. Unsurprisingly, Muslim visitors who value *Halal* cuisine highly for their self-concepts were more likely to experience *Halal* food anxiety. Pandemic travel anxiety and *Halal* food anxiety were positively correlated, whereas the psychological health of Muslim travellers was negatively correlated. Ambali and Bakar [138] discovered that religious belief, *Halal* exposure, exposure to the *Halal* logo, and health considerations determine Muslims’ awareness of *Halal* consumption.

Since major findings were highlighted in their research, Yama et al. [116] revealed that *Halal* certification could increase restaurant business performance. Their findings have also clarified the existence of partial mediation in the relationship between *Halal* certification and restaurant business performance due to ethical compliance. Yama et al. [116] found that businesses with *Halal* certification abide by the law and moral principles to ensure greater output. As a result, there are always justifications and demands for *Halal* food certification. The methodologies utilized to detect animal-derived components in meals and feeds utilizing molecular biology and nanotechnology were comprehensively reviewed by Ali et al. [139] in 2012. Many religious, regional, and state legislatures, as well as the food and feed industry, all need the authentication of animal-derived components. Verifying claimed components also aids in the suppression of unfair trade practices and the defence of customers’ trust, moral principles, and money [139]. Sharia laws defining *Halal* and haram meals, beverages, and items are included in Islamic beliefs [129]. Muslims, who make up the bulk of Indonesia’s customers, have come to rely on *Halal* and haram foods for their safety and well-being. The *Halal* company ought to implement this criterion [129].

### 4.3. How Are Halal-Based Sources for Biofertilizer Production Important for the Food–Water–Energy Nexus?

**Figure 3:**
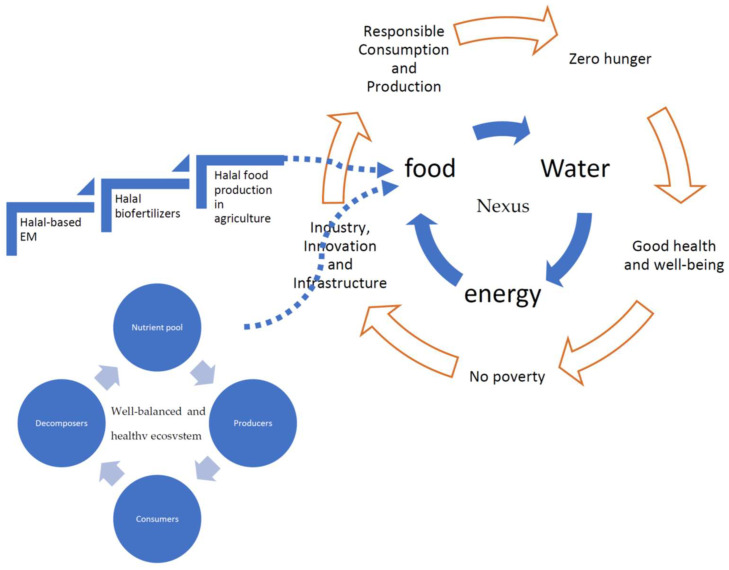
Connections of *Halal*-based sources for biofertilizer production for overall food chain supply for the food–water–energy Nexus.

The question of ‘How *Halal*-based sources for biofertilizer production is important for Nexus food-water-energy?’ is interesting since there is no absolute answer yet from all perspectives. When looking at the food–water–energy cycle (Figure 3), two interpretations can be offered. First, *Halal*-based sources for biofertilizer production for the overall food chain supply for the food–water–energy Nexus would have a positive impact on (a) industry innovation and infrastructure, (b) responsible consumption and production, (c) reducing hunger, (d) attaining good health and well-being, and (e) reducing poverty under the umbrella of the United Nations’ Sustainable Development Goals. It is considered that (a) and (b) are the most important and most closely related. Second, a well-balanced and healthy ecosystem could directly produce good food quantity and quality as natural resources can be recycled as long as the ecosystem is well maintained.

Microbial food components produced through biotechnology have a variety of applications in the food industry. New technological developments such as genetic editing might increase the efficiency of producing microbial products. Recently, microbial products and the innovative manufacturing techniques utilized to create them have confused some consumers. Many cultures have different food consumption habits depending on their beliefs and lifestyles. Muslims and other people who follow a specific religion or way of life are particularly interested in the microbiological components of their food. Microbiological products must correspond to the standards of the Islamic diet for Muslim cultures to thrive [197]. They thus require a few key standards for the production of bioproducts.

Hence, it is essential to evaluate a fermentation process from an Islamic viewpoint and determine the control points for *Halal* requirements to allay any worries about the meals prepared to utilize microbial metabolic products [197]. Some biotechnology companies have invested in adapting their production processes to comply with *Halal* regulations to participate in the *Halal* food sector. The market share of *Halal* microbial products is predicted to increase in the future [197].

Ali et al. [198] proposed a sustainable blockchain framework for the *Halal* food supply chain. While it is widely acknowledged that the blockchain could enhance supply chain integrity, its impacts on the *Halal* food supply chain are unknown. Based on five in-depth *Halal* food supply chain case studies, they revealed a practical framework for overcoming the challenges faced by the *Halal* food supply chain pertaining to blockchain implementation. They also indicated that the *Halal* food supply chain could gain a congruent and fresh perspective in inducing or superseding blockchain technology.

A theoretical viewpoint on Indonesia’s *Halal* food laws was offered by Azizah [99]. *Halal* food organizations significantly improve the quality of life for Muslims and other groups that buy their products. They found that since the Muslim community exclusively eats *Halal* food, legal regulation of *Halal* food is required in Indonesia. They recommended that regulatory agencies create strategies to prevent unethical behaviour successfully. The study has significant theoretical and practical ramifications for those involved in the Indonesian *Halal* food business. Mostafa [3] showed that the network of collaboration in the field of *Halal* cuisine is thin. According to Al-Shami and Abdullah [24], the *Halal* food business faces several difficulties in its management, promotion, and certification procedures.

From an economic standpoint, using *Halal*-certified biofertilizer can save cost and capital with fewer inputs. This is because EM technology may effectively address various agricultural issues, enhancing agricultural output [194]. Two significant aspects supporting the economic perspective were drawn from Karahalil’s [197] assessment of the fundamentals of generating microbial food components utilized in *Halal* cuisine. First, they noted that several biotechnology businesses have invested in adapting their production techniques to conform to *Halal* norms to compete in the *Halal* food industry. Second, they strongly anticipated that the proportion of *Halal* microbial products in the biotechnology sector would rise.

The frequency of insect pests and plant illnesses can be decreased through EM technology. This biotechnology can therefore aid in producing additional foods that can improve human health and wellness. As a result, EM technology can assist in increasing food output and resolving agricultural issues. Using EM technology for *Halal*-certified biofertilizers, environmental contamination can be reduced, and natural ecosystems can be safeguarded [3,17,99,164,165,166].

Sulaiman [17] tried to demonstrate that the sustainability of *Halal* food depends on preserving its natural resources, including agricultural goods such as fruits, vegetables, and animals. Rejeb et al. [26,167] found that the efficacy of *Halal* labelling, the deployment of quality-management systems, and the use of technology may all improve *Halal* processes and increase the nation’s economic performance. Changing the *Halal* food industry’s implementation of greening techniques and support for environmentalism might promote environmental sustainability. They offered crucial managemental recommendations for those who work in the *Halal* food industry, allowing them to have an overview of earlier research and utilize it as a standard for implementing sustainable practices in their *Halal* food businesses.

According to Bux et al. [59], the improvement in *Halal* certification and the blockchain tool could improve fair trade, ethical business, green animal breeding, and environmental economics, and thus sustainable development, even though several efforts in terms of innovation and cooperation by local authorities, industrial associations, and leading consumers are required. The global demand for *Halal* products continues to prove promising even if confronted by significant challenges in terms of certification, quality assurance, and inability to synchronize with the prevailing international standards [66]. Therefore, *Halal*-certified biofertilizer has significant implications for meeting the rising food demand caused by global Muslim population growth.

## 5. Concluding Remarks

In summary, the impacts of *Halal*-certified biofertilizers play a very important role in the societal well-being and economic growth and development in a Muslim country. Although *Halal* status is not a must for the marketing of food in the world, *Halal*-certified biofertilizer for the *Halal* certification of food products carries the greatest potential to enter the Muslim markets. Based on *Halal* and fertilizer publications found in the Scopus database, all 17 papers reviewed above provided no detailed information on the *Halal* status of organic and bio-organic biofertilizers inoculated with EMs. Hence, this review shows that studies on the *Halal* status of fertilizers (or biofertilizers) using EMs are greatly needed in future studies. This knowledge gap is interesting for further studies in the future. The impacts of *Halal*-certified biofertilizers play a very important role in a Muslim country’s societal well-being and economic growth and development. Although *Halal*-status is not a must for the marketing of food in the world, *Halal*-certified biofertilizer for the *Halal*-status of food products carries the greatest potential to enter the Muslim markets. Lastly, it is postulated that the successful usage of EMs using *Halal*-based sources for biofertilizer production will result in two major outcomes regarding the points of United Nations’ Sustainable Development Goals: # 9 (Industry, Innovation, and Infrastructure) and # 12 (Responsible Consumption and Production).

## Figures and Tables

**Table 1:** Title of publications including the keywords ‘Effective Microorganism’ (EM) and ‘fertilizer’ obtained from the Scopus database searched on 29 January 2023.

No.	Title of Project	Comment	Ref.
1	The liquid waste rubber industry makes liquid organic fertilizer with the addition of eceng gondok and EM4.	Is the liquid waste rubber industry *Halal*-based?	[174]
2	Effects of EM on yield and quality of cabbage	Are the EMs used *Halal*-based?	[175]
3	Effects of EM and bio-organic fertilizers on growth parameters and yield quality of *Nicotiana tabacum.*	Are the EMs and bio-organic fertilizers *Halal*-based?	[176]
4	Impact of EM and organic and mineral fertilizers on the growth and mycorrhizal colonization of *Fagus sylvatica* and *Quercus robur* seedlings.	Are the EMs and organic fertilizers *Halal*-based?	[177]
5	The proliferation of EM in vinasse and its application in the manufacture of livestock-waste-based fertilizers.	Are the EMs in vinasse used *Halal*-based?	[178]
6	Properties and applications of an organic fertilizer inoculated with EM.	Is organic fertilizer inoculated with *Halal*-based EMs?	[158]
7	Organic fertilizer potential using *Aspergillus niger*, *Pseudomonas putida* and EM from coconut water waste	Are EMs from coconut water waste *Halal*-based?	[179]
8	Design and Build of Information System on E-Commerce of organic waste decomposer and plant fertilizer EM-10	Is the Plant Fertilizer EM-10 *Halal*-based?	[180]
9	Effect of organic fertilizer and EM on growth, yield and quality of paddy-rice varieties	Are organic fertilizers and EMs *Halal*-based?	[181]
10	Foliar application of EM on pea as an alternative fertilizer	Are the EMs *Halal*-based?	[182]
11	Response of swiss chard and soil properties to co-application of different fertilizers with EM	Are the EMs *Halal*-based?	[183]
12	Effect of EM with liquid organic fertilizer on the growth of Pak Choi	Are the EMs with liquid organic fertilizer *Halal*-based?	[184]
13	Effective microorganism biochar-based fertilizer (EMBF) can improve the physiological properties of tobacco	Are the EMBFs *Halal*-based?	[185]
14	An appropriate coupling of water fertilizer with effective microorganisms biochar-based fertilizer (EMBF) application can improve the quality and yield of tobacco	Are the EMBFs *Halal*-based?	[186]
15	The combined application of nitrogenous fertilizers (NFs) and effective microorganisms (EM) improved growth, yield and nutrient accumulation in stevia plants	Are the Ems *Halal*-based?	[187]
16	Production of effective microorganisms using *Halal-based* sources	An excellent review. However, it did not specifically directly discuss *Halal*-based sources for biofertilizer production.	[188]
17	Recommended the most environmentally beneficial methods of using kitchen food waste, to produce natural, *Halal*, eco-friendly fertilizers.	An interesting study. However, the *Halal*-based EM sources in agriculture is not included in the discussion.	[189]

**Table 2:** The potential impacts of *Halal*-certified biofertilizers from a socio-economic perspective.

Potential Impacts
(a) Cater to the increasing *Halal* food demand due to expected Muslim population expansion;
(b) Contribute to the sustainable buying behaviour of consumers of *Halal* products in the future;
(c) Cater to the increasing number of Muslim travellers around the world;
(d) Increase the cost-effectiveness and marketability of food products, thus positively impacting national income;
(e) A positive driver for higher production of more *Halal* foods that can enhance food safety, human health, and well-being, thus increasing national income.

## Data Availability

Not applicable.

## Supplementary Materials

The following supporting information can be downloaded at https://www.mdpi.com/article/10.3390/foods12081702/s1: Table S1; article titles containing the keywords ‘*Halal*’ and Food’ were found using the Scopus database searched on 17 March 2023. A total of 483 papers were recorded. Of this total, 56 papers were categorized under ‘economics, econometrics and finance’ in the Scopus database. Table S2: article titles containing the of ‘*Halal*’ and Food’ were found using the Scopus database searched on 17 March 2023. A total of 483 papers were recorded. Of this number, 33 papers were categorized under ‘environmental sciences’ in the Scopus database. Table S3: article titles containing the keywords ‘*Halal*’ and Food’ were found using the Scopus database searched on 17 March 2023. A total of 483 papers were recorded. Of this number, 82 papers were categorized under ‘social sciences’ in the Scopus database.
