# Supplementary material for: Effective Microorganisms as Halal-Based Sources for Biofertilizer Production and Some Socio-Economic Insights: A Review
Source: Foods. 2023 Apr 19;12(8):1702. doi: 10.3390/foods12081702 (PMC10138028; doi:10.3390/foods12081702)
Supplement: Supplementary file 1 [file foods-12-01702-s001.zip › foods-2289110-supplementary.pdf]

foods

Review

The effective microorganisms as halal-based sources for biofertilizer production and some socio-economic insights: A review

Chee Kong Yap [1,*] and Khalid Awadh Al-Mutairi [2]

1 Department of Biology, Faculty of Science, Universiti Putra Malaysia, 43400 UPM Serdang, Selangor, Malaysia
2 Department of Biology, Faculty of Science, University of Tabuk, Tabuk P.O. Box 741, Saudi Arabia; kmutairi@ut.edu.sa
* Correspondence: yapckong@hotmail.com or yapchee@upm.edu.my

Table S1. With the keywords of 'Halal' and Food' must be found the article title using the Scopus database searched on 17 March 2023, a total of 483 papers was recorded. In this number, there was a total of 56 papers was categorized under 'economics, econometrics and finance' in the Scopus database.

N	Title	Yea	Perspec-	Country	Type	Re
1	Muslims' purchase intention of halal food: Evidence from Indonesia.	20	S-Ec	Indonesia	Re-	[1]
2	Systematic Literature Review of Halal Ethnic Foods Consumption; Health-Related Scientific; and Marketing Perspective.	20	Ec		Re-	[2]
3	Three decades of halal food scholarly publications: A PubMed bibliometric network analysis.	20	Ec		Re-	[3]
4	Enhanced knowledge sharing adoption model in the halal food industry.	20	Ec		Re-	[4]
5	Halal Food Certification, Financial Performance, and Sustainability Reporting: Comparative Study of Food and Beverage Firms in Malaysia and Indonesia.	20	Ec	Malaysia/ Indo-	Re-	[5]
6	What drives the Halal Food and Beverage Trade? A Gravity Model Investigation.	20	Ec		?	[6]
7	Halal Food Prediction Using the Similarity Graph Algorithms.	20	Ec		Re-	[7]
8	Analysis of Gen Z Muslims' Interpersonal Behavior in Halal Food Purchasing in Indonesia.	20	Ec	Indonesia	Re-	[8]
9	Education for young generations on halal food audit and certification: Challenges and future prospects.	20	Ec		?	[9]
10	IMPLEMENTATION AND IMPACT OF A HALAL FOOD STANDARD: AN EMPIRICAL STUDY OF MALAYSIA.	20	Ec	Malaysia	Re-	[1
11	Impact of Korea's Image on Attitudes, Norms, and Purchase Intentions of Halal Foods: A Comparison Between Indonesia and Malaysia.	20	Ec	Korea	Re-	[1
12	Muslim Consumers' Attitude Toward Non-Muslim's Halal Food Operators: Evidence from Malaysia.	20	Ec	Malaysia	Re-	[1
13	Country of Origin, Religiosity and Halal Awareness: A Case Study of Purchase Intention of Korean Food.	20	Ec	Korea	Re-	[1
14	The Effect of Halal Awareness on Purchase Intention of Halal Food: A Case Study in Indonesia.	20	Ec	Indonesia	Re-	[1
15	Examining the engagement of young consumers for religiously sanctioned food: the case of halal food in India.	20	S-Ec	India	Re-	[1
16	Determinants of the Intention to Consume Halal Food, Cosmetics and Pharmaceutical Products.	20	Ec		?	[1
17	Regulating good agriculture practices (Gaps) in enhancing sustainability of halal food: Malaysian experience.	20	Ec	Malaysia	Re-	[1
18	Safeguarding halal integrity through halal logistics adoption: A case of food manufacturers.	20	Ec		Re-	[1
19	The relationship between halal factors and purchase intention of food products as moderated by word-of-mouth communications.	20	Ec		Re-	[1
20	Clustering halal food consumers: A Twitter sentiment analysis.	20	Ec		Re-	[2
21	THE ROLE OF HALAL CERTIFICATION ON PURCHASE INTENTION TOWARDS FOOD PRODUCTS FROM MENA COUNTRIES: A SEM STUDY.	20	Ec	MENA?	?	[2
22	Commodified religion: The keys to halal food?	20	Ec		Re-	[2
23	Food certification: The relationships between organic and halal certification in Malaysian food retailing.	20	Ec	Malaysia	Re-	[2
24	MUSLIM CUSTOMER BEHAVIOR IN HALAL FOOD ONLINE PURCHASING.	20	Ec		Re-	[2
25	Kosher and halal regulations for nutraceutical and functional foods.		Ec		?	[2
26	Halal Food and Muslim Tourists.	20	Ec		?	[2
27	Does religiosity really matter in purchase intention of Halal certified packaged food products? A survey of Indian muslims consumers.	20	S-Ec	Malaysia In-	Re-	[2

28	Religious identity, community and religious minorities' search efforts for religiously sanctioned food: The case of halal food in non-Muslim majority mar-	20	Ec		Re-	[2
29	Halal food consumption as perceived by the non-muslim in Malaysia.	20	Ec	Malaysia	Re-	[2
30	Non-muslim consumers attitude and repurchase behaviour towards halal food: An application of buyer behaviour model.	20	Ec		Re-	[3
31	Studies on technology and halal food security: A review.	20	S-Ec		Re-	[3
32	Halal culinary tracking application at food souvenirs center based on analytical hierarchy process (AHP) method. (2017) Pertanika Journal of Social Sci-	20	S-Ec		Re-	[3
33	Supplier management in halal food supply chain: A preliminary case study.	20	S-Ec		Re-	[3
34	The roles of international entrepreneur orientation and geographical scope level to determine international performance: A case in the Malaysian Halal	20	Ec	Malaysia	Re-	[3
35	The concept of halal and halal food certification process in Malaysia: Issues and concerns.	20	Ec	Malaysia	?	[3
36	Sustainable food production: Insights of Malaysian halal small and medium sized enterprises.	20	Ec	Malaysia	?	[3
37	Halal food: Keeping pure and true	20	Ec		?	[3
38	Influence of the halal certification mark in food product advertisements in Malaysia.	20	Ec	Malaysia	Re-	[3
39	Application of halalan tayyiban in the standard reference for determining Malaysian halal food.	20	Ec	Malaysia	?	[3
40	Halal food supply chain knowledge and purchase intention.	20	Ec		?	[4
41	Case study 4: IKEA Malaysia and the halal food crisis.	20	Ec	Malaysia	Re-	[4
42	Halal food market and opportunities for logistics sector.	20	S-Ec		?	[4
43	Creating global competitiveness through culture and religion: An insight into the global strategic evolution and marketing of the halal food industry.	20	S-Ec		?	[4
44	Extenuating food integrity risk through supply chain integration: The case of halal food.	20	S-Ec		Re-	[4
45	The halal food industry. (2014) Handbook on Islam and Economic Life.	20	S-Ec		?	[4
46	Halal development and food exports: Evidence from Malaysia and the Middle Eastern Asian Countries.	20	Ec	Malaysia mid-	Re-	[4
47	Perceptions on Halal food certification in Hat Yai, Thailand.	20	Ec	Thailand	Re-	[4
48	The moderating effect of geographical scope on the relationship between managers' prior international knowledge and working experience and interna-	20	S-Ec	Malaysia	Re-	[4
49	Export barriers to Halal food processing small and medium enterprises (SMEs) in Malaysia.	20	S-Ec	Malaysia	Re-	[4
50	Business networks in halal food industries in Malaysia.	20	Ec	Malaysia	?	[5
51	Assessment of Consumers' confidence on Halal labelled manufactured food in Malaysia.	20	S-Ec	Malaysia	Re-	[5
52	Knowledge on halal food amongst food industry entrepreneurs in Malaysia.	20	S-Ec	Malaysia	?	[5
53	Halal food in New Zealand restaurants: An exploratory study.	20	Ec	NZ	Re-	[5
54	Halal food certification in China. (2019) The Routledge Handbook of Halal Hospitality and Islamic Tourism.	20	Ec	China	?	[5
55	Halal food: Cut-throat competition	20	Ec		?	[5
56	The demand for halal food among Muslim travellers in New Zealand.	20	Ec	NZ	?	[5

Note: Ec= Economy; S-Ec= Social Economy.

Table S2. With the keywords of 'Halal' and Food' must be found the article title using the Scopus database searched on 17 March 2023, a total of 483 papers was recorded. In this number, there was a total of 33 papers was categorized under 'environmental sciences' in the Scopus database.

N	Title	Ye		Coun	Type	R
1	The Embodiment of Muslim Intention Elements in Buying Halal Food Products: A Literature Review.	20	S-		Re-	[5
2	The Importance of Halal Certification for the Processed Food by SMEs to Increase Export Opportunities.	20	Ev		?	[5
3	Halal Food Sustainability between Certification and Blockchain: A Review.	20	S-		Re-	[5
4	Adoption of analytical technologies for verification of authenticity of halal foods–a review.	20	Ev		Re-	[6
5	Halal Food Prediction Using the Similarity Graph Algorithms. (2022) International Journal on Food System Dynamics, 13 (2), 165-173.	20	S-		Re-	[6
6	Identification non-timber forest products as a source of halal food for village communities around the forest. (2021) E3S Web of Conferences, 332, 04001.	20	Ev		Re-	[6
7	Young consumer awareness of halal food in Indonesia. (2021) E3S Web of Conferences, 332, 04004.	20	Ev	Indo-	Re-	[6
8	Halal and safety traceability of material, production, and serving of local food in Surabaya: A review. (2021) IOP Conference Series: Earth and Environmental Science, 924 (1),	20	Ev	Indo-	Re-	[6
9	Novel primer targeting the mitochondrial NADH dehydrogenase subunit 4 (ND4) and NADH dehydrogenase subunit 5 (ND5) for detection of porcine (Sus scrofa) DNA	20	Ev		Re-	[6
1	A generalization SPARQL federated query: An initial step towards machine-readable web of data for halal food products. (2021). IOP Conference Series: Earth and Environ-	20	Ev		Re-	[6
1	Philippine halal food export industry: life cycle, trends, and global outlook. (2021) E3S Web of Conferences, 316, 02044.	20	Ev	Phil-	?	[6
1	Halal certification impact on firms' corporate social responsibility disclosure: Evidence from the food & beverage sector in Italy.	20	S-	Italy	Re-	[6
1	Meat and meat-based products: Challenges and opportunities in halal food security.	20	Ev		?	[6
1	The involvement of young entrepreneurs in halal food products in Kelantan.	20	Ev	Ma-	Re-	[7
1	Comparison halal food regulation and practices to support halal tourism in Asia: A review.	20	Ev	Asia	Re-	[7
1	Integration of Halal Assurance System (HAS) in the Integrated Management System (IMS) to support food industry performance: A review.	20	Ev		Re-	[7
1	Bill of material analysis framework of food menu to increase raw material inventory efficiency and halal food inspection of culinary business.	20	Ev		?	[7
1	An investigation of the aspects affecting non-Muslim customers' purchase intention of halal food products in Jakarta, Indonesia.	20	Ev	Indo-	Re-	[7
1	Halal food performance and its influence on patron retention process at tourism destination.	20	Ev		Re-	[7
2	Characterization of aflatoxin b1 biomarkers as preference for antitoxin candidates for biosensors for halal food supply.	20	S-		Re-	[7
2	Determination of Consumer's Willingness to Pay for Halal Food.	20	Ev		Re-	[7
2	Concept of halal food development to support halal tourism: A review.	20	Ev		Re-	[7
2	Triplex PCR for halal authentication of processed food: Development and Characterization.	20	Ev		Re-	[6
2	An analysis of food waste from a flight catering business for sustainable food waste management: A case study of halal food production process.	20	Ev		Re-	[8
2	Halal food security: Forensic and laboratory management.	20	Ev		?	[8
2	An analysis of legal protection of halal and thoyib food consumers in court judgments.	20	Ev		Re-	[8

2	Design O2O recommendation system for halal food product (HFP) using rule-based user activities.	20	S-		Re-	[8
2	Halal food Certification in the UK and its impact on food businesses: A review in the context of the European Union.	20	Ev	EU	Re-	[8
2	Halal compliance decisions by food suppliers based on analytic hierarchy process.	20	S-		?	[8
3	Islamic tourism: Beyond than just halal food and a place for prayer.	20	Ev		?	[8
3	Increasing production and eliminating waste through lean tools and techniques for Halal food companies.	20	S-		Re-	[8
3	Environmental concern in managing halal food supply chain.	20	Ev		?	[8
3	Challenge of the Halal food certificate for food marketing: Implications from the local branding strategy of Thailand.	20	S-	Thai-	?	[8

Note: S-Ev= Social Environment; Ev= Environment.

Table S3. With the keywords of 'Halal' and Food' must be found the article title using the Scopus database searched on 17 March 2023, a total of 483 papers was recorded. In this number, there was a total of 82 papers was categorized under 'social sciences' in the Scopus database.

N	Title of paper	Ye	Per-	Country	Type	Re
1	Muslims' purchase intention of halal food: Evidence from Indonesia.	20	S-Ec	Indonesia	Research	[1]
2	Examining the engagement of young consumers for religiously sanctioned food: the case of halal food in India.	20	S-Ec	India	Research	[15
3	Does religiosity really matter in purchase intention of Halal certified packaged food products? A survey of Indian muslims consumers	20	S-Ec	Malaysia In-	Research	[27
4	Studies on technology and halal food security: A review.	20	S-Ec		Review	[31
5	Halal culinary tracking application at food souvenirs center based on analytical hierarchy process (AHP) method.	20	S-Ec		Research	[32
6	Supplier management in halal food supply chain: A preliminary case study.	20	S-Ec		Research	[33
7	Halal food market and opportunities for logistics sector.	20	S-Ec		Research	[42
8	Creating global competitiveness through culture and religion: An insight into the global strategic evolution and marketing of the halal	20	S-Ec		Research	[43
9	Extenuating food integrity risk through supply chain integration: The case of halal food.	20	S-Ec		Research	[44
10	The halal food industry. (2014) Handbook on Islam and Economic Life	20	S-Ec		Review	[45
11	The moderating effect of geographical scope on the relationship between managers' prior international knowledge and working ex-	20	S-Ec	Malaysia	Research	[48
12	Export barriers to Halal food processing small and medium enterprises (SMEs) in Malaysia.	20	S-Ec	Malaysia		[49
13	Assessment of Consumers' confidence on Halal labelled manufactured food in Malaysia.	20	S-Ec	Malaysia	Research	[51
14	Knowledge on halal food amongst food industry entrepreneurs in Malaysia.	20	S-Ec	Malaysia	Amongst Food Industry Entre-	[52
15	The Embodiment of Muslim Intention Elements in Buying Halal Food Products: A Literature Review.	20	S-Ev		Review	[57
16	Halal Food Sustainability between Certification and Blockchain: A Review.	20	S-Ev		Review	[59
17	Halal Food Prediction Using the Similarity Graph Algorithms.	20	S-Ev		Research	[61

18	Halal certification impact on firms' corporate social responsibility disclosure: Evidence from the food & beverage sector in Italy.	20	S-Ev	Italy	Research	[68
19	Characterization of aflatoxin b1 biomarkers as preference for antitoxin candidates for biosensors for halal food supply.	20	S-Ev		Research	[76
20	Design O2O recommendation system for halal food product (HFP) using rule-based user activities.	20	S-Ev		Research	[83
21	Halal compliance decisions by food suppliers based on analytic hierarchy process.	20	S-Ev		Research	[85
22	Increasing production and eliminating waste through lean tools and techniques for Halal food companies.	20	S-Ev		Research	[87
23	Challenge of the Halal food certificate for food marketing: Implications from the local branding strategy of Thailand.	20	S-Ev	Thailand	Research	[89
24	The characteristics of halal and non-halal food territories in multilayered mapping of Jakarta's Chinatown urban foodscape.	20	S	Indonesia	Research	[90
25	Halal food anxiety in a non-Muslim destination: the impact on the psychological well-being of Muslim travelers during the quarantine	20	S	China	Research	[91
26	"Food unites us… not anymore!?" Indonesian pilgrims eating kosher and halal in Jerusalem.	20	S	Israel	?	[92
27	Urban food environments and cultural adequacy: the (dis)assemblage of urban halal food environments in Muslim minority contexts.	20	S		Research	[93
28	Mining halal food search pathways down the Wikipedia's rabbit hole.	20	S			[94
29	Effect of alternative preservatives on the quality of rice cakes as halal food.	20	S		Research	[95
30	Online Traceability of Halal Food Information to Protect Muslim Consumers in the Cyber Era.	20	S		Research	[96
31	Exploring social media marketing strategies in small and medium enterprises (SMEs) of the halal food industry.	20	S		Research	[97
32	Desired Muslims: Neoliberalism, halal food production and the assemblage of Muslim expertise, service providers and labour in New	20	S	Brazil and	Research	[98
33	Stereotyping Halal Food and Eating Halal Food in a South Korean Context.	20	S	South Korea	Research	[99
34	Theoretical thought of Legal Regulation of Halal Food.	20	S			[10
35	Knowledge of halal Foods by University Students Enrolled in a Dietitian Training Course in Japan.	20	S	Japan	Research	[10
36	Halal for all: Geo spatial tourism of halal food stalls in Muslim minority tourist destinations.	20	S		Research	[10
37	Spatial and temporal changes in Halal food sales and consumption. A case study of the city of Dearborn, Michigan.	20	S	USA	Research	[10
38	Muslim tourists' purchase intention of halal food in Spain.	20	S	Spain	Research	[10
39	Blood, body and belonging: the geographies of halal food consumption in the UK.	20	S	UK	Research	[10
40	Halal food in the tourist destination and its importance for Muslim travellers.	20	S		Research	[10
41	Halal food tourism: Perceptions of relevance and viability for South African destinations.	20	S	South African	Research	[10
42	Extraction, and characterization of carotenoids from 11 allelopathic plant species as potential halal food colorants and active pharma-	20	S		Research	[10
43	Global halal food discourse on social media: a text mining approach.	20	S		Research	[10
44	Managing the symbolic power of halal meat in Swedish preschools: Food for thought in discussions on diversity.	20	S	Sweden	Research	[11
45	Perception of non-muslim manufacturers towards halal food supply chain in Malaysia.	20	S	Malaysia	Research	[11
46	The Promotional Language of the Halal Food Industry: Some Preliminary Findings.	20	S		Research	[11
47	"Siri is alligator halal?": Mobile apps, food practices, and religious authority among American Muslims.	20	S	USA	Research	[11
48	Optimal design for the Halal food logistics network.	20	S		Research	[11

49	Halal food security: Forensic and laboratory management.	20	S		Research	[11
50	Evaluation of the business excellent implementation among halal certified food manufacturers in Malaysia.	20	S	Malaysia	Research	[11
51	Halal food industry in Southeast Asia's Muslim majority countries: A reference for non-Muslim countries.	20	S	SEA	Research	[11
52	Impact of halal food certification on restaurant business in Indonesia: The mediating role of ethical compliance.	20	S	Indonesia	Research	[11
53	Halal food certification for improving the competitiveness of small and medium enterprises.	20	S		Research	[11
54	Halal virtual inspection requirements for food premise inspection process towards the virtualization of Malaysia Halal certification	20	S	Malaysia	Research	[12
55	Shariea As Taboo of Modern Law: Halal Food, Islamophobia, and China.	20	S	China	?	[12
56	Acculturation of halal food to the American food culture through immigration and globalization: A literature review.	20	S	USA	Review	[12
57	Managing demand growth for halal food. (2018) Fulfilling The Trust: 50 Years Of Shaping Muslim Religious Life In Singapore.	20	S	Singapore	?	[12
58	Controversial aspects of religious freedom in Spain and Europe: Halal food and casher.	20	S	Spain	?	[12
59	Halal chops and fascist cupcakes: On diversity and the weaponisation of food.	20	S		?	[12
60	Halal foods discourse and constructing Muslim identities in Japan.	20	S	Japan	?	[12
61	An adoption of halal food recognition system using mobile Radio Frequency Identification (RFID) and Near Field Communication	20	S		Research	[12
62	Legal conditions and controls for halal food Experience Indonesia.	20	S	Indonesia	?	[12
63	Halal food regulations in Malaysia: Concept and compliance.	20	S	Malaysia	?	[12
64	The usage of social media as an information seeking tool of Halal food products.	20	S		?	[13
65	The Concept of Halal Food in Sharia Perspective and Product Responsibility of Halal Industry.	20	S		?	[13
66	An attempt to apply actor-network theory to halal food production and supply in Japan.	20	S	Japan	Research	[13
67	The efficiency of halal processed food industry in Malaysia.	20	S	Malaysia	?	[13
68	Muslim food consumption in China: Between qingzhen and halal.	20	S	China	?	[13
69	Who owns halal?: Five international initiatives of halal food regulations.	20	S		?	[13
70	Application of halalan tayyiban in the standard reference for determining Malaysian halal food.	20	S	Malaysia	Research	[13
71	Islamic tourism: Beyond than just halal food and a place for prayer.	20	S		?	[13
72	Customer perceptions on Halal food quality towards their revisit intention: A case study on Chinese Muslim restaurants.	20	S	China	Research	[13
73	Strict Liability Food Law and Halal Slaughter.	20	S		?	[13
74	Rye bread and halal: Enregisterment of food practices in the primary classroom.	20	S		?	[14
75	Halal on the menu?: Contested food politics and French identity in fast-food.	20	S		?	[14
76	Halāl food and products in Malaysia: People's awareness and policy implications.	20	S	Malaysia	Research	[14
77	Species Authentication Methods in Foods and Feeds: The Present, Past, and Future of Halal Forensics.	20	S		Review	[14
78	Globalization: Explaining the dynamics and challenges of the halāl food surge.	20	S		?	[14
79	Halal food and tourism: Prospects and challenges.	20	S		?	[14

80	Illegal Labelling and Sales of Halal Meat and Food Products.	20	S		?	[14
81	Production of halal food: Practices of muslim small enterprises in Malaysia.	20	S	Malaysia	?	[14
82	Introduction Halal pizza: Food and culture in a busy world.	20	S		?	[14

Note: S= Social; S-Ec= Social economy; S-Ev= Social Environment.

References

[1] Febriandika, N.R., Wijaya, V., Hakim, L. Gen-Z. Muslims' purchase intention of halal food: Evidence from Indonesia. (2023) Innovative Marketing, 19(1), 3-25.

[2] Maqsood, R., Ayyub, R.M. Systematic Literature Review of Halal Ethnic Foods Consumption; Health-Related Scientific; and Marketing Perspective. (2023) Springer Proceedings in Business and Economics, pp. 203-226.

[3] Mostafa, M.M. Three decades of halal food scholarly publications: A PubMed bibliometric network analysis. (2022) International Journal of Consumer Studies, 46 (4), 058-1075.

[4] Samsi, S.Z M. Enhanced knowledge sharing adoption model in the halal food industry. (2022) Understanding, Implementing, and Evaluating Knowledge Management in Business Settings, pp. 83-105.

[5] Arieftiara, D., Nawir, J., Julianisa, I.A. Halal Food Certification, Financial Performance, and Sustainability Reporting: Comparative Study of Food and Beverage Firms in Malaysia and Indonesia. (2022) AgBioForum, 24 (2), 2-22.

[6] Hamid, B.A., Charoenchang, C., Dewandaru, G., Islam, M.U. What drives the Halal Food and Beverage Trade? A Gravity Model Investigation. (2022) Cuadernos de Economia, 45 (127), 91-201.

[7] Rakhmawati, N.A., Utomo, G.K., Indraswari, R., Susetyo, I.R. Halal Food Prediction Using the Similarity Graph Algorithms. (2022) International Journal on Food System Dynamics, 13 (2), 65-173.

[8] Bawono, A., Saputra, Y., Annur, F. Analysis of Gen Z Muslims' Interpersonal Behavior in Halal Food Purchasing in Indonesia. (2022) Journal of King Abdulaziz University, Islamic Economics, 35 (1), pp. 99-115.

[9] Hashmi, H.S., Ali, H. Education for young generations on halal food audit and certification: Challenges and future prospects. (2021) Contemporary Issues in Islamic Social Finance, pp. 273-292.

[10] Sari, D.P., Jaswir, I., Bin Haji Che Daud, Md.R. IMPLEMENTATION AND IMPACT OF A HALAL FOOD STANDARD: AN EMPIRICAL STUDY OF MALAYSIA. (2021) Journal of Islamic Monetary Economics and Finance, 7 (3), pp. 473-502.

[11] Yang, J.-J., Kim, D.-H., Lee, H.-Y. Impact of Korea's Image on Attitudes, Norms, and Purchase Intentions of Halal Foods: A Comparison Between Indonesia and Malaysia. (2021) Journal of Korea Trade, 25 (5), -18.

[12] Ariffin, S.K., Mohsin, A.M.A., Salamzadeh, Y. Muslim Consumers' Attitude Toward Non-Muslim's Halal Food Operators: Evidence from Malaysia. (2021) Eurasian Studies in Business and Economics, 11 (5), pp. 53-66.

[13] Astuti, Y., Asih, D. Country of Origin, Religiosity and Halal Awareness: A Case Study of Purchase Intention of Korean Food. (2021) Journal of Asian Finance, Economics and Business, 8 (4), pp. 0413-0421.

[14] Vizano, N.A., Khamaludin, K., Fahlevi, M. The Effect of Halal Awareness on Purchase Intention of Halal Food: A Case Study in Indonesia. (2021) Journal of Asian Finance, Economics and Business, 8 (4), pp. 441-453.

[15] Hassan, Y., Pandey, J. Examining the engagement of young consumers for religiously sanctioned food: the case of halal food in India. (2020) Young Consumers, 21 (2), pp. 211-232.

[16] SUDARSONO, H., NUGROHOWATI, R.N.I. Determinants of the Intention to Consume Halal Food, Cosmetics and Pharmaceutical Products. (2020) Journal of Asian Finance, Economics and Business, 7 (10), pp. 831-841.

[17] Sulaiman, S.S. Regulating good agriculture practices (Gaps) in enhancing sustainability of halal food: Malaysian experience. (2020) Malaysian Journal of Consumer and Family Economics, 24 (S2), pp. 32-45.

[18] Yunan, Y.S.M., Ali, M.H., Alam, S.S. Safeguarding halal integrity through halal logistics adoption: A case of food manufacturers. (2020) Institutions and Economies, 12 (3), 8-40.

[19] Hamzah, M.I., Othman, A.K., Rashid, W.E.W., Ngah, N.M.A. The relationship between halal factors and purchase intention of food products as moderated by word-of-mouth communications. (2020) International Journal of Business and Society, 21 (2), pp. 865-882.

[20] Mostafa, M.M. Clustering halal food consumers: A Twitter sentiment analysis. (2019) International Journal of Market Research, 61 (3), pp. 320-337.

[21] Perdana, F.F.P., Jan, M.T., Altunişik, R., Jaswir, I., Kartika, B. THE ROLE OF HALAL CERTIFICATION ON PURCHASE INTENTION TOWARDS FOOD PRODUCTS FROM MENA COUNTRIES: A SEM STUDY. (2019) Journal of Islamic Monetary Economics and Finance, 5 (1), pp. 63-88.

[22] Parlak, D. Commodified religion: The keys to halal food? (2019) The Routledge Handbook of Halal Hospitality and Islamic Tourism, pp. 326-335.

[23] Ibrahim, M.A., Michael Hall, C., Ballantine, P.W. Food certification: The relationships between organic and halal certification in Malaysian food retailing. (2019) The Routledge Handbook of Halal Hospitality and Islamic Tourism, pp. 264-277.

[24] Al-Banna, H. MUSLIM CUSTOMER BEHAVIOR IN HALAL FOOD ONLINE PURCHASING. (2019) Journal of Islamic Monetary Economics and Finance, 5 (3), pp. 517-540.

[25] Regenstein, J.M. Kosher and halal regulations for nutraceutical and functional foods. (2019) Nutraceutical and Functional Food Regulations in the United States and around the World, pp. 589-600.

[26] Haddad, R., Harahsheh, S., Harb, A. Halal Food and Muslim Tourists. (2019) The Routledge Handbook of Gastronomic Tourism, pp. 441-451.

[27] Khan, A., Azam, M.K., Arafat, M.Y. Does religiosity really matter in purchase intention of Halal certified packaged food products? A survey of Indian muslims consumers. (2019) Pertanika Journal of Social Sciences and Humanities, 27 (4), pp. 2383-2400.

[28] Mumuni, A.G., Veeck, A., Luqmani, M., Quraeshi, Z.A., Kamarulzaman, Y. Religious identity, community and religious minorities' search efforts for religiously sanctioned food: The case of halal food in non-Muslim majority markets. (2018) International Journal of Consumer Studies, 42 (6), pp. 586-598.

[29] Yunos, N., Mohamad, Z., Ghazali, M.A., Awang, M.D. Halal food consumption as perceived by the non-muslim in Malaysia. (2018) Malaysian Journal of Consumer and Family Economics, 21, 24-135.

[30] Damit, D.H.D.A., Harun, A., Martin, D., Shamsudin, A.S.B., Kassim, A.W.M. Non-muslim consumers attitude and repurchase behaviour towards halal food: An application of buyer behaviour model. (2018) WSEAS Transactions on Business and Economics, 15, pp. 413-422.

[31] Jalil, N.S., Norman, A.A., Hamid, S. Studies on technology and halal food security: A review. (2017) Pertanika Journal of Social Sciences and Humanities, 25 (December), pp. 225-239.

[32] Sucipto, S., Effendi, M., Khilmi, M.U., Kamal, M.A., Pinandito, A., Tolle, H. Halal culinary tracking application at food souvenirs center based on analytical hierarchy process (AHP) method. (2017) Pertanika Journal of Social Sciences and Humanities, 25 (December), pp. 51-64.
[33] Fujiwara, T. Supplier management in halal food supply chain: A preliminary case study. (2017) Pertanika Journal of Social Sciences and Humanities, 25 (December), pp. 25-38.
[34] Ismail, N.A. The roles of international entrepreneur orientation and geographical scope level to determine international performance: A case in the Malaysian Halal food industry. 2017) International Journal of Entrepreneurship, 21 (1), pp. 33-46.
[35] Asa, R.S., Azmi, I.M.A.G. The concept of halal and halal food certification process in Malaysia: Issues and concerns (2017) Malaysian Journal of Consumer and Family Economics, 20 (S1), pp. 38-50.
[36] Ali, M.H., Suleiman, N. Sustainable food production: Insights of Malaysian halal small and medium sized enterprises. (2016) International Journal of Production Economics, 181, pp. 303-314.
[37] Halal food: Keeping pure and true (2016) Economist (United Kingdom), 412 (9006).
[38] Hassan, S.H., Dann, S., Kamal, K.A.M., de Run, E.C. Influence of the halal certification mark in food product advertisements in Malaysia. (2016) The New Cultures of Food: Marketing Opportunities from Ethnic, Religious and Cultural Diversity, pp. 243-262.
[39] Arif, S., Sidek, S. Application of halalan tayyiban in the standard reference for determining Malaysian halal food (2015) Asian Social Science, 11 (17), 16-129.
[40] Yusoff, F.A.M., Yusof, R.N.R., Hussin, S.R. Halal food supply chain knowledge and purchase intention. (2015) International Journal of Economics and Management, 9 (Special Issue), 55-172.
[41] Mutum, D.S., Ghazali, E.M. Case study 4: IKEA Malaysia and the halal food crisis. (2014) Marketing Cases from Emerging Markets, 9783642368615, pp. 31-33.
[42] Yener, D. Halal food market and opportunities for logistics sector. (2014) Emerging Research on Islamic Marketing and Tourism in the Global Economy, 16-134.
[43] Camillo, A.A., Marques, J., Holt, S., Hu, J. Creating global competitiveness through culture and religion: An insight into the global strategic evolution and marketing of the halal food industry. (2014) Handbook of Research on Effective Marketing in Contemporary Globalism, pp. 335-347.
[44] Ali, M.H., Tan, K.H., Pawar, K., Makhbul, Z.M. Extenuating food integrity risk through supply chain integration: The case of halal food. (2014) Industrial Engineering and Management Systems, 13 (2), 54-162.
[45] Regenstein, J.M., Riaz, M.N., Chaudry, M., Regenstein, C.E. The halal food industry. (2014) Handbook on Islam and Economic Life, 75-194.
[46] Masron, T.A., Azman, N.H.N., Hassan, S.H. Halal development and food exports: Evidence from Malaysia and the Middle Eastern Asian Countries. (2014) Jurnal Ekonomi Malaysia, 48 (2), pp. 61-69.
[47] Abdul, M. Perceptions on Halal food certification in Hat Yai, Thailand. (2014) International Journal of Economics and Management, 8 (1), 78-194.
[48] Ismail, N.A., Mohamed, Z.A., Uli, J. The moderating effect of geographical scope on the relationship between managers' prior international knowledge and working experience and international performance in the Malaysian Halal Food Industry. (2013) Pertanika Journal of Social Science and Humanities, 21 (SPEC. ISSUE), 15-132.
[49] Ismail, M.M., Islam, M.S., Bakar, W.M.M.W. Export barriers to Halal food processing small and medium enterprises (SMEs) in Malaysia. (2013) Pertanika Journal of Social Sciences and Humanities, 21 (August), pp. 31-46.
[50] Idris, N.A.H. Business networks in halal food industries in Malaysia. (2013) Jurnal Ekonomi Malaysia, 47 (1), pp. 87-98.
[51] Rezai, G., Mohamed, Z., Shamsudin, M.N. Assessment of Consumers' confidence on Halal labelled manufactured food in Malaysia. (2012) Pertanika Journal of Social Science and Humanities, 20 (1), pp. 33-42.
[52] Rahman, A.H.A., Ahmad, W.I.W., Mohamad, M.Y., Ismail, Z. Knowledge on halal food amongst food industry entrepreneurs in Malaysia. (2011) Asian Social Science, 7 (12), pp. 216-221.
[53] Wan-Hassana, W.M., Awangb, K.W. Halal food in New Zealand restaurants: An exploratory study. (2009) International Journal of Economics and Management, 3 (2), pp. 385-402.
[54] Chen, N., Qi, S., Michael Hall, C. Halal food certification in China. (2019) The Routledge Handbook of Halal Hospitality and Islamic Tourism, pp. 249-263.
[55] Halal food: Cut-throat competition (2009) Economist, 392 (8649).
[56] Wan Hassan, M., Hall, C.M. The demand for halal food among Muslim travellers in New Zealand. (2003) Food Tourism Around The World, pp. 81-101.
[57] Wiyono, S.N., Deliana, Y., Wulandari, E., Kamarulzaman, N.H. The Embodiment of Muslim Intention Elements in Buying Halal Food Products: A Literature Review. (2022) Sustainability (Switzerland), 14 (20), 13163.
[58] Anggarkasih, M.G., Resma, P.S. The Importance of Halal Certification for the Processed Food by SMEs to Increase Export Opportunities. (2022) E3S Web of Conferences, 348, 00039.
[59] Bux, C., Varese, E., Amicarelli, V., Lombardi, M. Halal Food Sustainability between Certification and Blockchain: A Review. (2022) Sustainability (Switzerland), 14 (4), 2152.
[60] Kua, J.M., Azizi, M.M.F., Abdul Talib, M.A., Lau, H.Y. Adoption of analytical technologies for verification of authenticity of halal foods–a review. (2022) Food Additives and Contaminants - Part A, 39 (12), 1906-1932.
[61] Rakhmawati, N.A., Utomo, G.K., Indraswari, R., Susetyo, I.R. Halal Food Prediction Using the Similarity Graph Algorithms. (2022) International Journal on Food System Dynamics, 13 (2), 165-173.
[62] Latifah, S., Purwoko, A., Fachrudin, K.A., Siregar, J.W. Identification non-timber forest products as a source of halal food for village communities around the forest. (2021) E3S Web of Conferences, 332, 04001.
[63] Khaliqi, M., Pane, T.C. Young consumer awareness of halal food in Indonesia. (2021) E3S Web of Conferences, 332, 04004.
[64] Prayudanti, A.A., Sucipto, S. Halal and safety traceability of material, production, and serving of local food in Surabaya: A review. (2021) IOP Conference Series: Earth and Environmental Science, 924 (1), 012002.

[65] Kusnadi, J., Mahardita, P., Al-Awwaly, K.U., Arumingtyas, E.L. Novel primer targeting the mitochondrial NADH dehydrogenase subunit 4 (ND4) and NADH dehydrogenase subunit 5 (ND5) for detection of porcine (Sus scrofa) DNA fragments in food products for halal authentication. (2021) IOP Conference Series: Earth and Environmental Science, 924 (1), 012004.
[66] Hernawan, A., Sunarwidhi, A.L., Prasedya, E.S., Widyastuti, S. A generalization SPARQL federated query: An initial step towards machine-readable web of data for halal food products. (2021). IOP Conference Series: Earth and Environmental Science, 913 (1), 012040.
[67] de Castro, M.M., Mariano, R., Nuñez, C.L., Legaspi, A.P. Philippine halal food export industry: life cycle, trends, and global outlook. (2021) E3S Web of Conferences, 316, 02044.
[68] Secinaro, S., Radwan, M., Calandra, D., Biancone, P. Halal certification impact on firms' corporate social responsibility disclosure: Evidence from the food & beverage sector in Italy. (2021) Corporate Social Responsibility and Environmental Management, 28 (4), 1376-1385.
[69] Ruzulan, Z., Jamaludin, M.A., Ishak, A.H. Meat and meat-based products: Challenges and opportunities in halal food security. (2021) IOP Conference Series: Earth and Environmental Science, 756 (1), 012017.
[70] Nursalwani, M., Suhada, R.N.N., Sannasi, A.P., Amizi, A.M., Ariff, A.L.Z. The involvement of young entrepreneurs in halal food products in Kelantan. (2021) IOP Conference Series: Earth and Environmental Science, 756 (1), 012001.
[71] Nuraini, S., Sucipto. Comparison halal food regulation and practices to support halal tourism in Asia: A review. (2021) IOP Conference Series: Earth and Environmental Science, 733 (1), 012044.
[72] Puspaningtyas, S.D., Sucipto, S. Integration of Halal Assurance System (HAS) in the Integrated Management System (IMS) to support food industry performance: A review. (2021) IOP Conference Series: Earth and Environmental Science, 733 (1), 012045.
[73] Kamal, M.A., Effendi, F.D., Utomo, M.R., Sucipto, S., Santoso, I., Effendi, U. Bill of material analysis framework of food menu to increase raw material inventory efficiency and halal food inspection of culinary business. (2021) IOP Conference Series: Earth and Environmental Science, 733 (1), 012043.
[74] Arifin, A., Wyman, F., Shintawati, Hendijani, R.B. An investigation of the aspects affecting non-Muslim customers' purchase intention of halal food products in Jakarta, Indonesia. (2021) Future of Food: Journal on Food, Agriculture and Society, 9 (2), 1-13.
[75] Han, H., Lho, L.H., Raposo, A., Radic, A., Ngah, A.H. Halal food performance and its influence on patron retention process at tourism destination. (2021) International Journal of Environmental Research and Public Health, 18 (6), 3034, 1-15.
[76] Sukmanadi, M., Wahjuni, R.S. Characterization of aflatoxin b1 biomarkers as preference for antitoxin candidates for biosensors for halal food supply. (2020) Indian Journal of Forensic Medicine and Toxicology, 14 (4), 3162-3167.
[77] MadencI, A.B., Bayramoglu, Z., Türker, S., Agizan, K., EyIz, V. Determination of Consumer's Willingness to Pay for Halal Food. (2020) Journal of Tekirdag Agricultural Faculty, 17 (3), 346-356.
[78] Addina, F.N., Santoso, I., Sucipto. Concept of halal food development to support halal tourism: A review. (2020) IOP Conference Series: Earth and Environmental Science, 475 (1), 012053.
[79] Desriani, Sugiana, F.A., Widyowati, H., Suryani, Sukma, A., Wulandari, D., Warisman, M.A. Triplex PCR for halal authentication of processed food: Development and Characterization. (2020) IOP Conference Series: Earth and Environmental Science, 439 (1), 012047.
[80] Thamagasorn, M., Pharino, C. An analysis of food waste from a flight catering business for sustainable food waste management: A case study of halal food production process. (2019) Journal of Cleaner Production, 228, 845-855.
[81] Jaswir, I., Mirghani, M.E.S., Almansori, B.H., Octavianti, F. Halal food security: Forensic and laboratory management. (2019) Societal Dimensions of Environmental Science: Global Case Studies of Collaboration and Transformation, 117-128.
[82] Wiryani, F., Najih, M., Haris, A. An analysis of legal protection of halal and thoyib food consumers in court judgments. (2018) IOP Conference Series: Earth and Environmental Science, 175 (1), 012195.
[83] Nouh, R.M., Won-Jin, L., Jae-Dong, L. Design O2O recommendation system for halal food product (HFP) using rule-based user activities. (2017) Advanced Science Letters, 23 (10), 9491-9495.
[84] Fuseini, A. Halal food Certification in the UK and its impact on food businesses: A review in the context of the European Union. (2017) CAB Reviews: Perspectives in Agriculture, Veterinary Science, Nutrition and Natural Resources, 12, 007.
[85] Iberahim, H., Mustapha, N., Kamaruddin, R., Hamid, N.A., Samah, M.A.A. Halal compliance decisions by food suppliers based on analytic hierarchy process. (2016) Advanced Science Letters, 22 (12), 4509-4513.
[86] Mahamod, L.H., Yahya, W.N.A.W., Shamsuddin, W.N.W., Othman, R., Hashim, K.S.H.-Y., Ibrahim, M. Islamic tourism: Beyond than just halal food and a place for prayer. (2015) Advanced Science Letters, 21 (6), 2085-2088.
[87] Manzouri, M., Ab-Rahman, M.-N., Zain, C.R.C.M., Jamsari, E.A. Increasing production and eliminating waste through lean tools and techniques for Halal food companies. (2014) Sustainability (Switzerland), 6 (12), 9179-9204.
[88] Rahman, M.N.A., Manzouri, M., Che Mohd Zain, C.R. Environmental concern in managing halal food supply chain. (2013) American Journal of Environmental Sciences, 9 (3), 43-55.
[89] Kurokawa, K. Challenge of the Halal food certificate for food marketing: Implications from the local branding strategy of Thailand. (2011) Studies in Regional Science, 41 (4), 1045-1054.
[90] Komala, O.N., Ellisa, E., Andri Yatmo, Y. The characteristics of halal and non-halal food territories in multilayered mapping of Jakarta's Chinatown urban foodscape. (2022) A/Z ITU Journal of the Faculty of Architecture, 19 (1), pp. 119-135.
[91] Yousaf, S. Halal food anxiety in a non-Muslim destination: the impact on the psychological well-being of Muslim travelers during the quarantine period in China. (2022) Current Issues in Tourism, 25 (7), pp. 1136-1150.
[92] Lucking, M. "Food unites us… not anymore!?" Indonesian pilgrims eating kosher and halal in Jerusalem. (2022) Food, Culture and Society, 25 (4), pp. 699-711.
[93] de Araujo, S.H, Hamid, S.C., do Rego, A.G. Urban food environments and cultural adequacy: the (dis)assemblage of urban halal food environments in Muslim minority contexts. (2022) Food, Culture and Society, 25 (5), pp. 899-916.
[94] Mostafa, M.M. Mining halal food search pathways down the Wikipedia's rabbit hole. (2022) Food, Culture and Society, 25 (1), pp. 49-69.
[95] Oh, J., Kim, M.K. Effect of alternative preservatives on the quality of rice cakes as halal food. (2021) Foods, 10 (10), art. no. 2291.
[96] Azizah, S.N. Online Traceability of Halal Food Information to Protect Muslim Consumers in the Cyber Era. (2021) International Journal of Cyber Criminology, 15 (2), pp. 1-17.

[97] Shahkat Ali, M.S., Ahamat, A., Yas, R.B. Exploring social media marketing strategies in small and medium enterprises (SMEs) of the halal food industry. (2021) SEARCH Journal of Media and Communication Research, 2021 (Special Issue), pp. 157-168.
[98] Araujo, S.H.D. Desired Muslims: Neoliberalism, halal food production and the assemblage of Muslim expertise, service providers and labour in New Zealand and Brazil. (2021) Ethnicities, 21 (3), pp. 411-432.
[99] Nur Yasar, H. Stereotyping Halal Food and Eating Halal Food in a South Korean Context. (2021) European Journal of Korean Studies, 21 (1), pp. 259-290.
[100] Azizah, S.N. A Theoretical thought of Legal Regulation of Halal Food. (2021) International Journal of Criminal Justice Sciences, 16 (2), pp. 220-235.
[101] Yazawa, A., Kikuoka, T. Knowledge of halal Foods by University Students Enrolled in a Dietitian Training Course in Japan. (2021) Journal of Hospitality and Tourism Education, doi=10.1080%2f10963758.2021.1963741&partnerID=40&md5=284b3c50918d7d8bc30219fd7b0fbc65
[102] Perguna, L.A., Irawan, L.Y., Kurniawati, E., Triharini, S., Pahlevi, D.S., Febrianto, A.D. Halal for all: Geo spatial tourism of halal food stalls in Muslim minority tourist destinations. (2021) Geojournal of Tourism and Geosites, 36 (2 spl), pp. 715-720.
[103] Roodbar, S., Veeck, G. Spatial and temporal changes in Halal food sales and consumption. A case study of the city of Dearborn, Michigan. (2021) Journal of Cultural Geography, 38 (2), pp. 235-261.
[104] Pradana, M., Huertas-García, R., Marimon, F. Muslim tourists' purchase intention of halal food in Spain. (2021) Current Issues in Tourism, 24 (13), pp. 1814-1818.
[105] Isakjee, A., Carroll, B. Blood, body and belonging: the geographies of halal food consumption in the UK. (2021) Social and Cultural Geography, 22 (4), pp. 581-602.
[106] Mannaa, M.T. Halal food in the tourist destination and its importance for Muslim travellers. (2020) Current Issues in Tourism, 23 (17), pp. 2195-2206.
[107] Bhoola, S. Halal food tourism: Perceptions of relevance and viability for South African destinations. (2020) African Journal of Hospitality, Tourism and Leisure, 9 (3), pp. 288-301.
[108] Othman, R., Ramya, R., Azeman, M.A., Hassan, N.M., Kamoona, S. Extraction, and characterization of carotenoids from 11 allelopathic plant species as potential halal food colorants and active pharmaceutical ingredients. (2020) Journal of Pharmacy and Nutrition Sciences, 10 (1), pp. 19-24.
[109] Mostafa, M.M. Global halal food discourse on social media: a text mining approach (2020) Journal of International Communication, 26 (2), pp. 211-237.
[110] Stier, J., Sandstrom, M. Managing the symbolic power of halal meat in Swedish preschools: Food for thought in discussions on diversity. (2020) Journal of Intercultural Communication, 2020 (52), pp. 92-106.
[111]Ustadi, M.N., Osman, S., Rasi, R.Z. Perception of non-muslim manufacturers towards halal food supply chain in Malaysia. (2020) International Journal of Innovation, Creativity and Change, 10 (11), pp. 430-440.
[112] Ariffin, A., Fadzlullah, W.N.F.W.M. The Promotional Language of the Halal Food Industry: Some Preliminary Findings. (2019) Global Journal Al-Thaqafah, pp. 103-113.
[113] Fewkes, J.H. "Siri is alligator halal?": Mobile apps, food practices, and religious authority among American Muslims. (2019) Anthropological Perspectives on the Religious Uses of Mobile Apps, pp. 107-129.
[114] Kwag, S.I., Ko, Y.D. Optimal design for the Halal food logistics network. (2019) Transportation Research Part E: Logistics and Transportation Review, 128, pp. 212-228.
[115] Jaswir, I., Mirghani, M.E.S., Almansori, B.H., Octavianti, F. Halal food security: Forensic and laboratory management. (2019) Societal Dimensions of Environmental Science: Global Case Studies of Collaboration and Transformation, pp. 117-128.
[116] Masrom, N.R., Daut, B.A.T., Raja Mohd Rasi, R.Z., Niraula, B. Evaluation of the business excellent implementation among halal certified food manufacturers in Malaysia. (2019) Humanities and Social Sciences Reviews, 7 (2), pp. 220-225.
[117] Widyantoro, S., Arsyad, R., Fathoni, M. Halal food industry in Southeast Asia's Muslim majority countries: A reference for non-Muslim countries. (2019) Intellectual Discourse, 27, pp. 767-781.
[118] Yama, A., Jermsittiparsert, K., Saregar, A. Impact of halal food certification on restaurant business in Indonesia: The mediating role of ethical compliance. (2019) International Journal of Innovation, Creativity and Change, 9 (7), pp. 306-322.
[119] Ratnasari, R.T., Gunawan, S., Possumah, B.T., Rusmita, S.A., Widiastuti, T., Herianingrum, S. Halal food certification for improving the competitiveness of small and medium enterprises (2019) Opcion, 35 (Special Issue 22), pp. 510-525.
[120] Sulaiman, M.Z.M., Noordin, N., Md Noor, N.L., Suhaimi, A.I.H., Isa, W.A.R.W.M. Halal virtual inspection requirements for food premise inspection process towards the virtualization of Malaysia Halal certification system. (2018) Proceedings - International Conference on Information and Communication Technology for the Muslim World 2018, ICT4M 2018, art. no. 8567088, pp. 12-17.
[121] Erie, M.S. Shariea As Taboo of Modern Law: Halal Food, Islamophobia, and China. (2018) Journal of Law and Religion, 33 (3), pp. 390-420.
[122] Halawa, A. Acculturation of halal food to the American food culture through immigration and globalization: A literature review. (2018) Journal of Ethnic and Cultural Studies, 5 (2), pp. 53-63.
[123] Suratty, D.H. Managing demand growth for halal food. (2018) Fulfilling The Trust: 50 Years Of Shaping Muslim Religious Life In Singapore, pp. 199-219.
[124] Garcia, A.L. Controversial aspects of religious freedom in Spain and Europe: Halal food and casher. (2017) Estudios Constitucionales, 15 (2), pp. 331-364.
[125] Hussein, S. Halal chops and fascist cupcakes: On diversity and the weaponisation of food. (2017) Meanjin, 76 (1), pp. 124-129.
[126] Ono, J. Halal foods discourse and constructing Muslim identities in Japan. (2017) Feeding Japan: The Cultural and Political Issues of Dependency and Risk, pp. 313-337.
[127] Khosravi, M., Karbasi, M., Shah, A., Brohi, I.A., Ali, N.I. An adoption of halal food recognition system using mobile Radio Frequency Identification (RFID) and Near Field Communication (NFC). (2017) Proceedings - 6th International Conference on Information and Communication Technology for the Muslim World, ICT4M 2016, art. no. 7814879, pp. 70-75.
[128] Lopes, A.B. Legal conditions and controls for halal food Experience Indonesia. (2017) Ahkam: Jurnal Ilmu Syariah, 17 (1), pp. 241-258.

[129] Ismail, C.T., Rahman, A.A., Abdullah, N.A. Halal food regulations in Malaysia: Concept and compliance. (2017) Man in India, 97 (24), pp. 291-300.
[130] Yusoff, S.Z., Mohamad, N., Ghazali, M.F., Abdullah, N.H., Syed Azmy, S.N.M. The usage of social media as an information seeking tool of Halal food products. (2016) International Journal of Applied Linguistics and English Literature, 5 (6), pp. 245-248.
[131] Ali, M. The Concept of Halal Food in Sharia Perspective and Product Responsibility of Halal Industry. (2016) Ahkam: Jurnal Ilmu Syariah, 16 (2), pp. 291-306.
[132] Wataru, N. An attempt to apply actor-network theory to halal food production and supply in Japan. (2016) Japanese Journal of Human Geography, 68 (4), pp. 421-441.
[133] Mohd Noor, M.A., Rizki Moi, M., Abdul Kader, R. The efficiency of halal processed food industry in Malaysia. (2016) Global Journal Al-Thaqafah, 6 (1), pp. 37-46.
[134] Sai, Y., Fischer, J. Muslim food consumption in China: Between qingzhen and halal. (2015) Halal Matters: Islam, Politics and Markets in Global Perspective, pp. 160-174.
[135] Bergeaud-Blackler, F. Who owns halal?: Five international initiatives of halal food regulations. (2015) Halal Matters: Islam, Politics and Markets in Global Perspective, pp. 192-197.
[136] Arif, S., Sidek, S. Application of halalan tayyiban in the standard reference for determining Malaysian halal food. (2015) Asian Social Science, 11 (17), pp. 116-129.
[137] Mahamod, L.H., Yahya, W.N.A.W., Shamsuddin, W.N.W., Othman, R., Hashim, K.S.H.-Y., Ibrahim, M. Islamic tourism: Beyond than just halal food and a place for prayer. (2015) Advanced Science Letters, 21 (6), pp. 2085-2088.
[138] Rejab, N.H., Ruhadi, N.R.A.N., Arsat, A., Jamil, J., Hassan, H. Customer perceptions on Halal food quality towards their revisit intention: A case study on Chinese Muslim restaurants. (2015) Theory and Practice in Hospitality and Tourism Research - Proceedings of the 2nd International Hospitality and Tourism Conference 2014, pp. 381-384.
[139] Pointing, J. Strict Liability Food Law and Halal Slaughter. (2014) Journal of Criminal Law, 78 (5), pp. 387-391.
[140] Karrebak, M.S. Rye bread and halal: Enregisterment of food practices in the primary classroom. (2014) Language and Communication, 34 (1), pp. 17-34.
[141] Wright, W., Annes, A. Halal on the menu?: Contested food politics and French identity in fast-food. (2013) Journal of Rural Studies, 32, pp. 388-399.
[142] Ambali, A.R., Bakar, A.N. Halāl food and products in Malaysia: People's awareness and policy implications. (2013) Intellectual Discourse, 21 (1), pp. 7-32.
[143] Ali, M.E., Kashif, M., Uddin, K., Hashim, U., Mustafa, S., Che Man, Y.B. Species Authentication Methods in Foods and Feeds: The Present, Past, and Future of Halal Forensics. (2012) Food Analytical Methods, 5 (5), pp. 935-955.
[144] Adams, I.A. Globalization: Explaining the dynamics and challenges of the halāl food surge. (2011) Intellectual Discourse, 19 (1), pp. 123-145.
[145] Bon, M., Hussain, M. Halal food and tourism: Prospects and challenges. (2010) Bridging Tourism Theory and Practice, 2, pp. 47-59.
[146] Pointing, J., Teinaz, Y., Shafi, S. Illegal Labelling and Sales of Halal Meat and Food Products. (2008) Journal of Criminal Law, 72 (3), pp. 206-213.
[147] Othman, R., Mohamad Zailani, S.H., Ahmad, Z.A. Production of halal food: Practices of muslim small enterprises in Malaysia. (2008) International Journal of Interdisciplinary Social Sciences, 3 (9), pp. 69-76.
[148] James, R. Introduction Halal pizza: Food and culture in a busy world. (2004) Australian Journal of Anthropology, 15 (1), pp. 1-11.
